# Enzymatic Antioxidant Defense and Polymorphic Changes in Male Infertility

**DOI:** 10.3390/antiox11050817

**Published:** 2022-04-22

**Authors:** Jędrzej Baszyński, Piotr Kamiński, Maria Bogdzińska, Sławomir Mroczkowski, Marek Szymański, Karolina Wasilow, Emilia Stanek, Karolina Hołderna-Bona, Sylwia Brodzka, Rafał Bilski, Halyna Tkachenko, Natalia Kurhaluk, Tomasz Stuczyński, Małgorzata Lorek, Alina Woźniak

**Affiliations:** 1 Department of Ecology and Environmental Protection, Department of Medical Biology and Biochemistry, Collegium Medicum in Bydgoszcz, Nicolaus Copernicus University in Toruń, M. Skłodowska-Curie St. 9, PL 85-094 Bydgoszcz, Poland; jedrzej.baszynski@cm.umk.pl (J.B.); emilia.stanek@cm.umk.pl (E.S.); karhol89@gmail.com (K.H.-B.); sylwia_florczak89@wp.pl (S.B.); malgorzatalorek1@gmail.com (M.L.); 2 Department of Biotechnology, Faculty of Biological Sciences, Institute of Biological Sciences, University of Zielona Góra, Prof. Z. Szafran St. 1, PL 65-516 Zielona Góra, Poland; 3 Department of Genetics and Animal Breeding, Faculty of Animal Breeding and Biology, UTP University of Science and Technology in Bydgoszcz, Hetmańska St. 33, PL 85-039 Bydgoszcz, Poland; bogdzinska@utp.edu.pl (M.B.); mroslav@utp.edu.pl (S.M.); 4 Department of Obstetrics, Female Pathology and Oncological Gynecology, University Hospital No. 2, Collegium Medicum in Bydgoszcz, Nicolaus Copernicus University in Toruń, Ujejski St. 75, PL 85-168 Bydgoszcz, Poland; marek.szymanski@cm.umk.pl; 5 NZOZ Medical Center Co., Waleniowa St. 24, PL 85-435 Bydgoszcz, Poland; karolina.wasilow@cm.umk.pl; 6 Family Medicine Clinic, University Hospital No. 2, Collegium Medicum in Bydgoszcz, Nicolaus Copernicus University in Toruń, Ujejski St. 75, PL 85-168 Bydgoszcz, Poland; 7 Department of Medical Biology and Biochemistry, Collegium Medicum in Bydgoszcz, Nicolaus Copernicus University in Toruń, M. Karłowicz St. 24, PL 85-092 Bydgoszcz, Poland; rafal.bilski@cm.umk.pl (R.B.); or alina-wozniak@wp.pl (A.W.); 8 Department of Biology, Institute of Biology and Earth Sciences, Pomeranian University in Słupsk, K. Arciszewski St. 22 B, PL 76-200 Słupsk, Poland; halyna.tkachenko@apsl.edu.pl (H.T.); natalia.kurhaluk@apsl.edu.pl (N.K.); 9 Department of Soil Structure, Institute of Soil and Plant Cultivation-Government Scientific Institute, Czartoryskich St. 8, PL 24-100 Puławy, Poland; ts@iung.pulawy.pl or; 10 Faculty of Mathematics Informatics and Landscape Architecture, The John Paul II Catholic University of Lublin, Konstantynów 1 H, PL 20-708 Lublin, Poland

**Keywords:** male infertility, oxidative stress, chemical elements, enzymatic antioxidants, genetic polymorphism, *IL-4*

## Abstract

The intensification of oxidative stress and destabilization of the antioxidative defenses of an organism is a consequence of many environmental factors. We considered aspects conditioning male reproductive potential and the functionality of enzymatic antioxidative mechanisms, i.e., superoxide dismutase (SOD), catalase (CAT), glutathione peroxidase (GPx) and glutathione reductase (GR), and their correlations with Li, Be, B, Na, Mg, Al, P, K, Ca, Ti, V, Cr, Mn, Fe, Co, Ni, Cu, Zn, As, Se, Sr, Mo, Ag, Cd, Sn, Sb, Ba, Hg, Tl, Pb, and malondialdehyde (MDA), as well as genetic polymorphism *IL-4*v.*C589T* (rs2243250) in men with infertility (*n* = 76). A healthy normozoospermic control (*n* = 87) was also used. We assessed the impact of negative changes driven by oxidative stress on enzymatic antioxidative mechanisms as well as the role of MDA in the overall process. On this basis, we infer connections between disturbances in enzymatic antioxidative defense and reproductive potential. Based on a molecular analysis of the polymorphism of gene *IL-4*v.*C589T* (rs2243250) (chromosome 5) (PCR-RFLP), we considered the relationships among particular genotypes with the possibility of occurrence of male infertility. Concentrations of chemical elements were measured in the blood. The activity of antioxidants and MDA levels were measured in serum. In the infertile group, higher GPx activity was noted (6.56 nmoL·min^−1^·mL^−1^, control: 4.31 nmoL·min^−1^·mL^−1^; *p* = 0.004), while GR achieved a greater level in the control (17.74 nmoL·min^−1^·mL^−1^, infertile: 15.97 nmoL·min^−1^·mL^−1^, *p* = 0.043), which implies diversified efficiency of the first and second lines of defense. The polymorphism of *IL-4*v.*C589T* (rs2243250) was not directly connected with infertility because there were not any differences in the frequency of genotypes between the infertile and control group (*p* = 0.578). An analysis of genotypes *CC* and *TT* (polymorphism *IL-4*v.*C589T* (rs2243250)) indicated numerous correlations between antioxidants, chemical elements and MDA. Therefore, chemical economy, antioxidative defense and genetic conditions are connected and jointly shape male reproductive potential. Chemical elements influence antioxidative defense and male fertility; the most important modulators appeared to be Na, Ba, Al and B. The polymorphism of gene *IL-4*v.*C589T* (rs2243250) has a limited influence on antioxidative defense and the metabolism of chemical elements.

## 1. Introduction

Infertility is defined as the inability to induce pregnancy in a period of twelve months of regular intercourse without contraception. The fact that multiple factors are associated with male infertility make it difficult to identify the cause of the condition [1]. McLeod was the first to suggest that oxidative stress may play a role in the etiology of defective functionality of sperm (1943; [2]). He observed that in an oxygenated medium, human sperm suddenly lost their motility. The effect could be reversed by the antioxidative activity of catalase. The notion that sperm, as a result of its own activities, may generate reactive oxygen species (ROS) was introduced by Tosic and Walton (1946; [2]). Oxidative stress is a lack of balance between the generation of ROS and their effective elimination. Damage may result when the level of ROS or other free radicals is considerably increased while the antioxidative abilities of an organism are decreased (i.e., a lack of pro-antioxidative equilibrium) [3,4]. Increased ROS levels appear in 25–80% of infertile men. Infertility is usually further connected with low levels of antioxidants in semen compared to fertile men. Also, in idiopathic cases, excess ROS is a frequent phenomenon [3,5]. The coexistence of oxidative stress and male infertility is additionally augmented by a high intensity of cellular divisions and mitochondrial oxygen consumption in testis tissue, as well as a high percentage of unsaturated fatty acids in the membrane structure of sperm [6]. On the other hand, small amounts of ROS are necessary to finalize sperm maturation and obtain fertility. Therefore, increments in ROS accompany the process of capacitation, while small amounts of hydrogen peroxide or nitric oxide favor the transition of sperm to a state of hyperactivation, which marks the beginning of the acrosomal reaction. These processes are fundamental for the maintenance of male reproductive potential. However, uncontrolled increments in ROS cause damage to the sperm structure, lipid peroxidation of cellular membranes, fragmentation of sperm DNA and reduction in vitality and motility, as well as a general decrease in sperm quality [5].

There are many potential sources of ROS which may be linked to male infertility, like activated leucocytes from inflammation processes, immature sperm with abnormal morphologies and defective spermatogenesis. Also varicocele, cryptorchidism or testicle torsion increase the risk of ROS overproduction [4]. In addition to these internal sources of oxidative stress, external sources remaining under particular patient control (lifestyle, addictions, occupational exposure) are equally important; each of these factors may lead to increased ROS in semen [4]. Sperm is particularly susceptible to oxidative stress resulting from the consumption of large amounts of polyunsaturated fatty acids, or from small amounts of intracellular antioxidative enzymes and limited DNA repair ability. Considering this susceptibility, a high percentage of idiopathic cases of infertility are attributed to elevated levels of ROS in semen and decreased antioxidative potential. ROS cause infertility by damaging sperm DNA or by initiating lipid peroxidation of membrane structures, which negatively impacts both sperm motility and potential to combine with oocytes [7]. In the initial stages of development, male reproductive cells generate small amounts of ROS that regulate the number of sperm (stimulation of apoptosis and proliferation of spermatogonia), ensuring proper condensation of sperm chromatin, as well as serving as signal molecules. Therefore it is fundamental to maintain ROS on a physiological level, as excessive ROS generation is a precursor to oxidative stress [8]. Sperm is also subjected to oxidative damage due to its relatively small content of cytoplasm (i.e., a reservoir of antioxidative enzymes in somatic cells that constitute the first line of defense). Sperm mitochondria are potential sources of ROS. However, a certain amount of physiological ROS is necessary for sperm maturation [9]. Effective antioxidative defense is particularly important, considering the multitude of environmental pollutants and factors resulting in ROS generation (ionizing and nonionizing radiation, UV, increased ozone concentration, sulfur dioxide, nitrogen oxide, environmental contamination such as asbestos, dioxins, furans, aromatic polycyclic hydrocarbons, etc.) [10,11].

The existence of several types of superoxide dismutase is fundamental for enzyme functionality as well as cellular signalization processes. SOD is crucial for the inhibition of oxidative deactivation of nitric oxide, which inhibits the generation of oxidant ONOO^−^ and counteracts dysfunctions of the endothelium and mitochondria [12]. In these processes, Cu activates SOD1 and SOD3 post-transcriptionally, while Mn insertion takes place in newly synthesized SOD2 in cooperation with mitochondrial transporters. Lead perturbs SOD activity via the formation of complexes with SOD1, impeding access and destabilizing the balance of beneficial zinc and copper [13]. Enzymes make up about 3% of the proteins contained in seminal plasma. SOD, CAT, GPx, and GR have been found to exhibit relatively high activity in the fractions of ejaculate rich in sperm [14]. Fertility impairments are often accompanied by decreased total antioxidant capacity, as well as decreased non-protein antioxidants and potentially even higher protein antioxidant activity. This effect may result from an imbalance between protein and non-protein antioxidants when the downgrading of non-protein molecules triggers an increase of antioxidative proteins. However weaker GPx activity usually appears in severe asthenozoospermia, oligozoospermia and teratozoospermia compared with normozoospermia [15]. Also, seminal plasma levels of GPx, SOD, and CAT tend to be lower in infertile men compared to fertile controls. CAT is an enzyme which plays an important role in the protection of sperm against ROS, as H_2_O_2_ as well as superoxide anions constitute the main causes of reduced sperm quality and functionality. CAT molecules convert millions of hydrogen peroxide molecules into water and oxygen each second, and elevated CAT levels can be achieved by various antioxidant therapies, leading to improved sperm parameters and decreased lipid peroxidation. Beneficial effects appear to be augmented by the synergistic actions of different antioxidants, e.g., SOD and CAT [16]. CAT has a prostatic origin, since it can be detected even after vasectomy. It is also considered a beneficial factor for prolonged sperm survival in artificial insemination techniques. Acatalasemia is a condition caused by autosomal recessive gene mutation which manifests in significant enzyme deficiencies (less than 10% of normal activity). This condition appears to be connected with various chronic diseases, including fertility disorders [17].

Gene sequences encoding SOD, CAT or GPx are phylogenetically conserved. However, the activity of enzymes may undergo modulation as a consequence of single nucleotide polymorphisms affecting their antioxidative potential, increasing the risk of male fertility [18]. For example, it is possible that in the case of the single nucleotide polymorphism *SOD2* rs4880 (Val16Ala–C to T substitution in mitochondria targeting sequence, resulting in the substitution of valine by alanine), infertile men with genotype *CC* show lower SOD activity than *TT* genotype carriers [19]. In the case of *CAT C-262T*, genotype *CT* probably favors fertility, while *CC* doubles the risk of infertility [20]. Nevertheless, the exact role of single nucleotide polymorphisms in sperm antioxidative defense modulation has not yet been sufficiently explained [18]. Experiments with animal models have confirmed that a lack, deficiency, or perturbed activity of SOD have serious systemic consequences, e.g., uncontrolled ROS accumulation [12,21,22,23,24]. Another fundamental process in sperm antioxidative defense modulation is the reduction of H_2_O_2_, for which CAT is a crucial factor [10,25,26]. Rindler et al. (2013), using a mouse model, proved that a high-fat diet (60% from fat) favors an intensification of lipid peroxidation and overproduction of H_2_O_2_, but also initiates a defensive enzymatic antioxidative mechanism, i.e., increased CAT activity [27]. Numerous studies based on animal models or cultures of human cells have unambiguously confirmed the role of CAT in cellular defensive mechanisms against oxidative stress [28]. Sperm antioxidative defenses are also consolidated by GPx with selenocysteine in its active side reducing hydrogen peroxide. This enzyme has various isoforms, i.e., nuclear, cytosolic and mitochondrial; the latter mainly preserves sperm functions [29]. Hsieh et al. (2006) noted that the antioxidative role of GPx systems in response to lipoperoxidation has a positive effect on sperm functionality, general semen quality and indirectly on the crucial stages of sperm maturation and gaining the ability to fertilize oocytes. Simultaneously, disorders in GPx activity have negative impacts on male reproductive potential (reduced GPx activity in semen accompanies male infertility) [30]. Chabory et al. (2009) postulated that mammalian epididymis constitute an area of intensive GPx5 expression (unconventional type devoid of selenocysteine that could be secreted to epididymis, where it interacts with sperm). Therefore, it is possible that GPx5 is a key factor conditioning sperm maturation in epididymis, despite the fact that other isoforms of GPx containing selenium GPx1,3,4 were also detected in this area [31].

Sperm antioxidative defenses are also consolidated by MDA, which, as a breakdown product generated during lipoperoxidation, is one of basic factors determining the intensity of this process (i.e., it indicates an intensification of oxidative stress) [32]. Thus, MDA level can provide information about the extent of oxidative stress, and it may be assumed that sperm are affected by this phenomenon when symptoms of greatly enhanced sperm ROS amount, increased seminal MDA concentrations and decreased seminal plasma total antioxidant capacity are present [33]. Since polyunsaturated fatty acids are responsible for regulating sperm membrane fluidity, they are also susceptible to the cascade effect of lipid peroxidation, resulting in the formation of MDA, as well as other lipid aldehydes with high reactivity like 4-hydroxynonenal [32]. Subsequently, this phenomenon perturbs fundamental parameters connected with sperm quality causing impairments in membrane integrity, motility and general metabolism [34]. Considering male infertility as a problem connected with oxidative stress (as many as 40% of infertile men show increased ROS levels in semen, which is often accompanied by lowered antioxidative capacity), the application of therapies based on antioxidants seems relevant. An antioxidative-rich diet, accordingly dosed, may improve sperm quality as well as lessening oxidative damage [8,35].

In this study, we analyzed the relationships between concentrations of Li, Be, B, Na, Mg, Al, P, K, Ca, Ti, V, Cr, Mn, Fe, Co, Ni, Cu, Zn, As, Se, Sr, Mo, Ag, Cd, Sn, Sb, Ba, Hg, Tl, Pb, and enzymatic antioxidants SOD, CAT, GPx, and GR in men with fertility disorders and healthy controls. Correlations with MDA were also included. Therefore, we assessed the impact of the negative changes driven by oxidative stress on the functionality of enzymatic antioxidative mechanisms, as well as the role of MDA in process globally. On this basis, we reached conclusions about connections between disturbances in enzymatic antioxidative defenses and general reproductive potential. Using molecular analyses of the polymorphisms of the genes located on chromosome 5 (*IL-4*v.*C589T* (rs2243250), we considered the relationships of particular genotypes with the possibility of occurrence of male fertility disturbances. By establishing relationships between perturbations in the antioxidative barrier, the influence of chemical elements, MDA and the role of genetic polymorphisms, we revealed the significance of environmental factors which participated in oxidative stress, thereby shaping male reproductive potential. The dependencies analyzed in this research have not been studied via such an approach so far. As such, this research is innovative in nature.

## 2. Materials and Methods

### 2.1. Seminological Studies

The group of patients with infertility consisted of 76 men with various fertility disorders, as confirmed via seminological tests [36]. Semen abnormalities (quantitative and qualitative) applied to oligozoospermia, asthenozoospermia, azoospermia, teratozoospermia, necrozoospermia, combined oligozoospermia- asthenozoospermia-teratozoospermia OAT II, OAT III, cryptozoospermia, polyzoospermia, cryptoteratozoospermia, leukospermia or combined disorders. The control group consisted of 87 men with normozoospermia (no abnormalities in a seminal examination [36]). The majority of the men who qualified for the infertile group came from Bydgoszcz and the surrounding area (Central Poland), and were undergoing infertility treatment at the GENESIS clinic in Bydgoszcz (Medical Center, Bydgoszcz, Poland). The study area encompassed the Cuyavian region [37]. Therefore, the examination of infertile men from Bydgoszcz allowed us to analyze the impact of environmental stressors on male reproductive condition. All participants stated their conscious and voluntary agreement to participate in the study, received information on the objectives and completed a questionnaire. The collection of biological material and the seminological tests were conducted by qualified workers at the GENESIS clinic (immediately after collection of semen sample). The authors of this paper also analyzed semen samples. Evaluations applied to morphological parameters such as volume, time of liquefaction, sperm density (number per milliliter of ejaculate), motility (with specification of types of movement), presence of agglutination, presence of leukocytes, and sperm morphology.

On this basis, normozoospermia or specific seminological abnormalities were diagnosed, which allowed us to place patients in either the infertile or control group. In addition to semen (>1.5 mL), full blood samples (about 15 mL) and serum samples (1.5 mL) were also collected. Serum was separated from the blood by centrifugation at 3500 cycles per minute for 10 min. Serum and semen were stored in Eppendorf type tubes. Full blood and serum samples were conserved at −80 °C (New Brunswick Scientific-86 Co. Inc. 175 Ultra Low Temperature Freezer, Freshwater Blvd., Enfield, Connecticut, Edison, NJ, USA), while semen was kept in liquid nitrogen (−196 °C).

Study participants were men who voluntarily attended a private clinic for infertility treatment to obtain a seminological evaluation. Men that fulfilled the norms of the WHO (2010) [36] regarding semen parameters were qualified to join the control group (normozoospermia), while those with diagnosed impairments in any of parameters (in reference to norms of WHO 2010 [36]) were qualified to join the infertile group. We did not apply any specific recommendations regarding the age of participants. We also did not introduce any specified parameters that might influence exclusion from the study. The average age of infertile men was 31.29 years, while in the control group it was 29.02 years (*p* = 0.05). Surveys that were voluntarily filled out by each participant provided information about their health and potential environmental or genetic burdens (in the survey, there were questions about age, residence, kind of work, exposure to stress factors—physical or chemical, lifestyle, diet, innate or acquired diseases, diseases in family members, and medication use. Participants received complete information about the nature of the study project and expressed voluntary agreement to participate.

### 2.2. Methods Used for Seminological Analysis

Seminological analyses were based on macro- and microscopic analysis of ejaculate to verify parameters such as semen volume, time of liquefaction, sperm density (count per milliliter of ejaculate), motility (regarding types of movement), presence of agglutination, presence of leukocytes, and percentage of pathological forms. After comparison with the WHO reference values (2010) [36], subjects were assigned to either the infertile or control group. The criteria of normozoospermia (control group) in our study were defined on the basis of the following reference values: semen volume (≥1.5 mL), time of liquefaction (<60 min), sperm density (≥15 million·mL^−1^), sperm motility–types of movement A, B, C, D (progressive motility ≥32%), agglutination (lack of agglutinates and sperm aggregates), presence of leukocytes (<1 million·mL^−1^), and morphology (≥4% of correct forms). Deviation from these norms constituted a basis for classification in the infertile group, in which we singled out the following diagnoses: asthenozoospermia (*n* = 15; 19.74%), azoospermia (*n* = 10; 13.16%), cryptozoospermia (*n* = 3; 3.95%), necrozoospermia (*n* = 1; 1.32%), oligoasthenoteratozoospermia OAT (*n* = 14; 18.42%), oligozoospermia (*n* = 7; 9.21%), polyzoospermia (*n* = 4; 5.26%), and combined disorders (*n* = 22; 28.95%).

### 2.3. Standard Semen Evaluation

All of the tested parameters were determined immediately after liquefaction or within one hour of ejaculation at room temperature.

### 2.4. Macroscopic Evaluation of Semen

Volume (mL): measurement was carried out in a measuring cylinder with a conical base. Reaction (pH): The measurement was made with a Spezial-Indicator paper indicator (pH range 7.2–9.7; Macherey-Nagel GmgH & Co., Düren, Nordrhein-Westfalen, Germany) by putting a drop of semen on it. After 30 s, the color obtained at the point of contact was compared with the colors in the template provided by the manufacturer. Measurements were made within one hour of ejaculation.

### 2.5. Microscopic Evaluation of Semen

A Makler sperm counting chamber (Sefi Medical Instruments, Haifa, Israel) inserted into a light microscope (Carl Zeiss Jena, Jena, Germany) with Ph2 phase contrast was used for microscopic evaluation of semen. A small drop of semen, well mixed by pipetting, was placed in the center of a Makler chamber, and the chamber was covered with a coverslip. The preparations were then viewed under a magnification of 200× at room temperature.

The microscopic evaluations focused on the following parameters:

1. Concentration and motility of sperm in semen:

Assessments in the Makler’s chamber were performed in accordance with the principles of using a hemocytometer. During observations, the entire surface of the preparation was first viewed, and then sperm within 10 squares arranged along the diagonal mesh were counted (in the case of oligozoospermia, concentrations were in the hundreds of thousands per mL, while lower sperm counts were observed in all areas of the chamber-100 fields). The type of movement was then assessed, distinguishing among fast (type A) and slow (type B) forward (type A) and slow (type B) sperm, non-progressive (type C) sperm and non-moving (type D) sperm.

2. Morphological characteristics:

Sperm were observed under an immersion microscope with a magnification of 1000 times (100× objective) after prior staining with a semen smear using the Schorr method:

Reagents: Haemotoxylin Papanicolaou No. 1; ammonium alcohol (95 mL 75% ethanol + 5 mL 25% (250 mL·L^−1^) ammonium hydroxide); and Schorr’s solution.

Preparation of smear: about 20 µL of sample was applied (50–100 × 10^6^ sperms·mL^−1^) to a glass slide, smeared, and allowed to dry.

Dyeing Procedure:

– the slide was rinsed with running water (12–15 dips);
– the smear was immersed in Haemotoxylin Papanicolaou No. 1 for 1–2 min;
– the slide was rinsed with running water (12–15 dips);
– the smear was immersed in ammonium alcohol 5 times for 5 min;
– the slide was rinsed with running water (12–15 dips);
– the smear was immersed in 50% ethanol (500 mL·L^−1^) for 5 min;
– the smear was immersed in Schorr’s dye for 3–5 min;
– the smear was immersed in 50% ethanol (500 mL·L^−1^) for 5 min;
– the smear was immersed in 75% ethanol (750 mL·L^−1^) for 5 min;
– the smear was immersed in 95% ethanol (950 mL·L^−1^) for 5 min;
– the smear was immersed in absolute ethanol 2 times for 5 min;
– the smear was immersed in xylene (dimethylbenzene) twice for 5 min.

Taking into account the criteria included in the Kruger classification [38], after staining with the Schorr method, sperm were considered normal if they exhibited:

1. An oval head with a regular and clean contour (front view) or resembling a flattened pear (in profile). Sperm were divided by staining into two parts—the front, less saturated, covering the nuclear zone (acrosome), and the rear, with more compact nuclear mass. The length of the head was 4–5 µm, the width was 2–3 µm, and the length to width ratio was 1.5 to 2.
2. The intermediate part of the withers comprised a simple thickening in the extension of its long axis, and may have been surrounded by a small cytoplasmic residue, 4–5 µm in length.
3. The main part of the twine was thin, tapering regularly and measuring 8–9 times the length of the head. In our analyses of spermiograms, the ejaculate was treated as normal (normozoospermia) when the volume of sperm ranged from 2 to 6 mL, the number of sperm ranged from 20 to 250 million mL^−1^, the percentage of sperm in the progressive movement (type A + B) was not less than 50% and the percentage of normal sperm was at least 30% (abnormal maximum 70%).

Research was undertaken following the Guidelines of the European Union Council and the current laws in Poland, according to the Bioethical Commission of the Collegium Medicum of Nicolaus Copernicus University in Toruń, Poland. Samples were collected under permit No. KB 427/2014, and No. KB 365/2015).

### 2.6. Chemical Element Analysis

The concentrations of Li, Be, B, Na, Mg, Al, P, K, Ca, Ti, V, Cr, Mn, Fe, Co, Ni, Cu, Zn, As, Se, Sr, Mo, Ag, Cd, Sn, Sb, Ba, Hg, Tl, and Pb in the blood of participants were analyzed. To this end, the preparatory stage comprised blood mineralization. Quantitative analyses of chemical elements were conducted with mass spectrometry with inductively coupled plasma (ICP-MS). This method is very specific and makes it possible to determine chemical element concentrations with relatively small amounts of material. Before examination, sample mineralization was conducted. Full blood was transferred to calibrated tubes made of borosilicate glass (25 mL). After mineralization, redistilled water was added to the solution (up to 8 mL), which was then transferred to polyethylene containers. Samples were prepared in clean hood class 100 to prevent contamination with atmospheric particles.

### 2.7. Digestion

Digestion was conducted with the microwave method (Discovery SP-D, CEM Corporation, Matthews, NC, USA). Subsequently, samples were tested for vestigial minerals with an Agilent 7500CE ICP-MS (quadrupole mass spectrometry system using helium collision mode, Agilent Technologies, Inc, Headquarters, Santa Clara, CA, USA). Before digestion, all samples were thoroughly vortexed to achieve homogeneous matrix for digestion. Samples were immediately pipetted to prevent settling before extraction. Then, 250 µL samples were dosed into glass vessels designed for microwave digestion and washed with acid. Blind reagents were prepared by adding deionized water instead of samples. Nest, 300 μL concentrated nitric acid (HNO_3_) (Ultrex purity, Fisher, Thermo Fisher Scientific, Waltham, MA, USA), 200 μL concentrated hydrochloric acid (HCl) (Ultrex, Fisher, Thermo Fisher Scientific, Waltham, MA, USA) and 100 μL unstable 30% hydrogen peroxide solution (H_2_O_2_) (Ultrex, Fisher, Thermo Fisher Scientific, Waltham, MA, USA) were added to each phial with serum samples. Deionized water was added to obtain a final volume of 2.0 mL. Samples were closed tightly, placed in microwave cooker and digested according to serum digesting protocol. At the end of digestion, all samples were removed and left to cool to room temperature. In a clean hood, samples were transferred to polypropylene tubes (acid washed; capacity 15 mL) enriched with multi-elemental internal patterns to obtain final concentrations of 10 ng·mL^−1^ of indium, scandium, and praseodymium, and diluted to the final volume with deionized (DI) H_2_O. Samples were preserved in a monitor refrigerator at a nominal temperature of 4 °C until further testing. The mineralization method was applied to all chemical elements; no separate mineralization was performed.

The effectiveness of the applied method and good recoveries were experimentally confirmed in reference to mercury. Microwave digestion with concentrated nitric acid and hydrogen peroxide was used to break down the analyzed material. To 2 mL of 30% hydrogen peroxide was added 8 mL of 69–70% nitric acid (Baker Instra Analyzed, J.T. Baker Chemical Company, Avantor, Phillipsburg, NJ, USA). Subsequently, samples were exposed to microwaves for 5 min at 190 °C (time of escalation: 5 min), and finally, 5 min at 210 °C (time of escalation: 5 min) to ensure the complete dissolution of organic matter. Digested solutions were transferred to single calibrated tubes and mixed with deionized water (0.05 µS·cm^−1^) to achieve a final volume of 50 mL.

### 2.8. Quantitative Analyses

Quantitative analyses of trace elements were based on the ICP-MS technique (inductively coupled plasma mass spectrometry). The Agilent Technologies 7500CE ISP-MS apparatus is equipped with micro-mist nebulizer, double-pass spray chamber cooled with the Peltier method, and a peristaltic pump. Argon 5.0 (99.999% cleanness) was used as the carrier gas. The apparatus is also equipped with a torch with reduction system (shield torch) that eliminates interference. The vacuum system is made up of rotary pump and turbo-molecular pump. Quadrupol is mass separator with hyperbolical sticks. The detector operates in two modes, i.e., digital and analog, which makes it possible to work in nine grades of volume. All analyses were performed in the presence of 45Sc, 89Y, and 159Tb as internal patterns to minimize the effect of the matrices and ensure long-term stability. This procedure was also conducted for blind samples to detect pollution. Simultaneously for each sample series, certified reference material was used (NCS ZC73016 chicken) from the China National Analysis Center for Iron and Steel to meet the relevant quality control requirements. For this material, recoveries were obtained in the range of 90–110% and uncertainty of measurements was established at <10%. To measure the concentrations of chemical elements, and ICP-MS Agilent 7500 CE was used. It was equipped with nebulizer (micro-mist) and a mist chamber which was thermoelectrically cooled via the Peltier effect (double-pass). Additionally, it featured a reaction chamber ORS (octopole reaction system) to eliminate polyatomic interference as well as interference from doubly charged ions. In the reaction chamber, we used hydrogen 6.0 and helium 6.0 (99.9999% cleanness) as reaction gases to eliminate interference. Highly clean argon 5.0 (99,999% cleanness) was used as the transferring gas (pressure 200 bar). Samples were passed along at a speed of 0.01–0.5 rpm. Additionally, each analysis was performed with ^45^Sc, ^89^Y, and ^159^Tb as internal patterns to maintain the stability of the apparatus, as well as to minimize matrix effects.

### 2.9. Mineralization

The mineralization of biological samples in a microwave oven in hermetically closed teflon-coated containers prevents the volatile loss of mercury Hg. After mineralization, containers are cooled to room temperature, and then, the solution is transferred to a regular vessel. Problems with mercury may arise from the fact that in the presence of nitric acid, reduction of mercury may occur. Additionally, adsorption of mercury on hydrophobic surfaces of teflon-coated vessels used for mineralization may create a problem or cause a memory effect which is typical for mercury. In this respect, 20 tubes were checked by adding mercury to assess the influence of mineralization in the oven on the recovery efficiency. For the sake of comparison, we analyzed samples with additional patterns which were not mineralized; other elements were not analyzed and the amount of acid and final sample volume remained the same. Therefore, it is obvious that mineralization does not result in a loss of analytes; recoveries were very good, i.e., in samples conserved with EDTA, they were close to 100%, while in the case of samples with lithium heparin, they were around 90%. Mineralization occurred in teflon-coated containers which, after mineralization, were cooled to prevent losses. The low pH of the solution after mineralization and quantitative transfer to the final volume effectively prevented Hg loss. The problem of the influence of pH on Hg loss was examined by Rosain and Wai (1973) [39]. They found that the process of mercury mineralization does not result in the loss of this element. The first calibration pattern on the curve was 50 ng·L^−1^. The recovery of mercury from samples fortified in this way was almost 100% in the case of tubes with EDTA, whilst in the case of heparin, it was over 90%. For samples analyzed without mineralization, recoveries are lower, as nebulizer and plasma processes may interfere with non-mineralized EDTA and heparin. Patterns without matrices were also mineralized to obtain final concentrations after transfer to 25 mL of around 10 ng·L^−1^, 25 ng·L^−1^ and 50 ng·L^−1^. Again, the obtained recoveries were satisfactory. During each measurement, the same conditions were applied. In order to monitor the conditions, an internal pattern was applied. The apparatus receiving the signal from the internal pattern calculated the current results while eliminating the influence of environmental factors (changes in flow caused by decrease of opening diameter in the cone due to the sedimentation of calcium).

### 2.10. Calibration and Reference Materials

The calibration curves were produced using the weight method. A mixture of nitric acid (V) and water served as the blind sample, and was exposed under the mineralization conditions along with the other samples. Multi-elemental patterns were used with the appropriate conditions for ICP examinations. Because patterns were multi-elemental, it was not possible to prepare separate curves (with different ranges) for each chemical element. Certified reference materials were used as quality controls (QC) in ICP-MS analyses of serum samples. We applied reference materials Seronorm Trace Elements Serum L-1 and Serum L-2, which were marked at the beginning and the end of each group consisted of 20 samples. Simultaneously, for each sample series, certified reference material was used (NCS ZC73016 chicken) from the China National Analysis Center for Iron and Steel to fulfill quality control requirements. For this material, obtained recoveries were in the range of 90–110% and measurement uncertainty was established at 10%.

### 2.11. Enzymatic Antioxidants

Examinations of SOD activity in serum were conducted with a commercially available set manufactured by Cayman Chemicals Co. Ltd. (Superoxide Dismutase Assay Kit, Item No. 706002, Cayman Chemical Company, Ann Arbor, MI, USA). The procedure was based on tetrazolium salt, which makes it possible to detect superoxide radicals generated by xanthine oxidase and hypoxanthine. One unit of SOD represented the amount of enzyme necessary to perform dismutation of 50% of superoxide radicals. The set simultaneously measured the activity of several types of SOD (Cu/Zn, Mn, Fe-SOD). An examination of CAT activity in serum was conducted with a commercially available set manufactured by Cayman Chemicals Co. Ltd. (Catalase Assay Kit, Item No. 707002, Cayman Chemical Company, Ann Arbor, MI, USA). The procedure was based on the peroxidative propriety of a catalase-enzyme reaction with methanol in the presence of an optimal concentration of hydrogen peroxide. The produced formaldehyde was measured colorimetrically with 4-amino-3-hydrazino-5-mercapto-1,2,4-triazole as a chromogen. This formed cyclic compounds with aldehydes which, after oxidation, changed in appearance, from colorless to purple. An examination of GPx activity in serum was conducted with a commercially available set manufactured by Cayman Chemicals Co. Ltd. (Glutathione Peroxidase Assay Kit, Item No. 703102, Cayman Chemical Company, Ann Arbor, MI, USA). This activity was measured via an indirect method, i.e., a coupled reaction with glutathione reductase (GR). Oxygenated glutathione (GSSG), produced after the reduction of hydrogen peroxide by GPx, was restored to its reduced form by GR and NADPH. The oxidation of NADPH to NADP^+^ is connected with reduced absorbance at a wavelength of 340 nm. The scale of this downgrade is directly proportional to the activity of GPx. An examination of GR activity in serum was conducted with a commercially available set manufactured by Cayman Chemicals Co. Ltd. (Glutathione Reductase Assay Kit, Item No. 703202, Cayman Chemical Company, Ann Arbor, MI, USA). The procedure was based on a measurement of the oxidation efficiency of NADPH. The oxidation of NADPH to NADP^+^ downgrades absorbance at 340 nm, which is directly proportional to the activity of GR in the sample.

### 2.12. Lipid Peroxidation Intensity

An examination of the MDA (lipid peroxidation marker) level in serum was conducted with a commercially available set manufactured by Cayman Chemicals Co. Ltd. (TBARS Assay Kit, Item No. 10009055, Cayman Chemical Company, Ann Arbor, MI, USA). The procedure was based on measurement of the concentration of substances reacting with thiobarbituric acid (TBARS), which is a reflection of lipid peroxidation intensity. The presence of more unsaturated lipids causes an increment in TBARS value. Adduct MDA-TBA is produced in the reaction of MDA with TBA at high temperature (90–100 °C) and in conditions of acidic pH. The procedure was performed colorimetrically.

### 2.13. Molecular Analysis

The applied DNA isolation kit (Epicentre an Illumina Co.; Cat. No. MCD85201, Madison, WI, USA) consisted of Red Cell Lysis Solution, Tissue & Cell Lysis Solution (as well as T&C Lysis Solution 2×), MPC Protein Precipitation Reagent, RNase A, Proteinase K, and TE buffer (10 mM Tris-HCl, pH 7.5, 1 mM EDTA). After isolation, DNA was evaluated regarding its usefulness for further analysis. Quantities (ng·µL^−1^) and pureness (A260/A280) were measured with NanoDrop 2000 (Thermo Scientific, Thermo Fisher Scientific, Waltham, MA, USA). In this respect, 2 µL of DNA in TE buffer was adequate for effective measurements (the measurement apparatus was first calibrated with 2 µL of pure TE buffer-blank). DNA concentration in the range of 50–150 ng·µL^−1^ and pureness in the range of 1.6–1.9. were considered adequate (values were established based on optimization and the available literature; [40,41]). Horizontal electrophoresis was used (horizontally placed gel covered with 1×TBE buffer). The advantages of this method are the lack of risk of leakage of electrolytes and easy transfer of heat [42]. In our study, we used 2% agarose gels. The PCR reaction mix was prepared in sterile conditions (UVP UV3 HEPA PCR Workstation chamber). The order of reagents was established during optimization, guaranteeing optimal activity, as well as minimizing the use of unspecific products. To micro-tubes containing 1 µL of previously isolated DNA was transferred 19 µL of reaction mix with sterile pipettes and single tips (final reaction volume: 20 µL). These were placed in a thermal cycler, where a programmed protocol was performed. Applied starters, PCR reaction conditions (predenaturation, denaturation, annealing, elongation, and additional elongation) as well as the conditions of digestion with restrictive enzymes for respective polymorphism were established based of the available literature [43].

### 2.14. Polymorphism of Gene IL-4v.C589T (rs2243250) 

In the analysis of the polymorphism of the *IL-4*v.*C589T* (rs2243250) gene starter, forward 5’-TAAACTTGGGAGAACATGGT-3’ and reverse 5’-TGGGGAAAGATAGAGTAATA-3’ were used (Sigma-Aldrich, St. Louis, MO, USA). Before application, they were diluted with TE buffer according to the Sigma Technical Datasheet (forward: 1281 µL of TE; reverse: 1163 µL of TE). PCR reaction products were sectioned through electrophoresis with an apparatus designed for horizontal electrophoresis (MS Major Science MP-300V, GenBiotech, Roseti, C1427 BWB, Buenos Aires, Argentina) at 110 V for about 60 min on 2% agarose gel. In the final step, each sample was digested with a restrictive enzyme (*AvaII*) to obtain restriction fragments with a specific length (PCR-RFLP). Based on the restriction fragments image, it was possible to distinguish particular genotypes (heterozygote, wild type homozygote, and mutated homozygote). The conditions of electrophoresis after digestion were about 90 min on 2% agarose gel at a voltage of 110 V.

### 2.15. Statistical Analysis

Statistical analyses were performed with STATISTICA (v.13, StatSoft, Cracov, Poland) and Microsoft Office Excel 2021 (v.2108, Microsoft, Redmond, WA, USA). Regarding quantitative parameters, the results are displayed as minimum and maximum values, quartiles (Q1, Q3), medians, arithmetical averages and standard deviations. Data were analyzed for normal distribution. Those that did not exhibit normal distribution were analyzed with non-parametric tests (U-Mann-Whitney, Spearman rank test). Normally distributed data were compared with a Student-*t* test. Correlations were analyzed with Spearman rank correlation tests. The coefficient of significance was set at α < 0.05 and statistical significance at *p* < 0.05 [44,45,46].

## 3. Results

### 3.1. Activity of Enzymatic Antioxidants 

**Figure 1:**
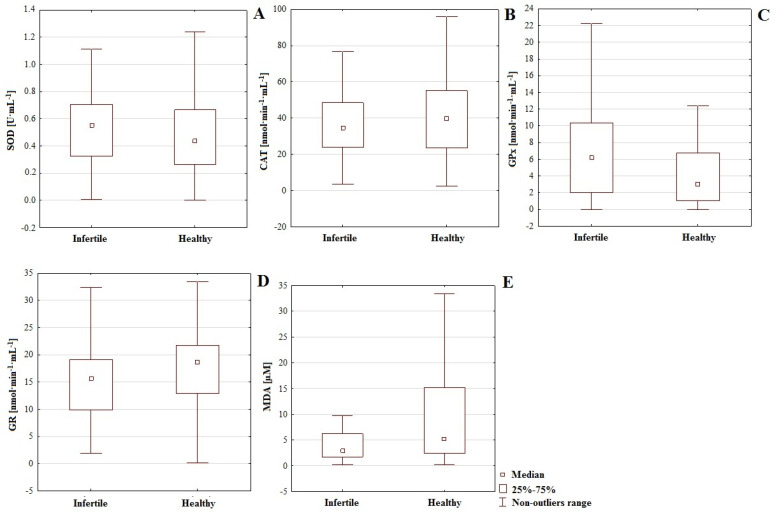
Activity of superoxide dismutase SOD (**A**), catalase CAT (**B**), glutathione peroxidase GPx (**C**), and glutathione reductase GR (**D**); concentration of malondialdehyde MDA (**E**) in healthy controls and men with fertility disorders.

Significant differences were observed in the activity of the enzymatic antioxidants applied to GR, that exhibited higher activity in men from the healthy group. For GPx, higher values of enzymatic activity appeared in the infertile group. For SOD and CAT, there were no significant differences. However, the activity of SOD was higher in the infertile group, while for CAT, activity was greater in the healthy group (Table 1, Figure 1). Analysis of the enzymatic defense components in particular groups revealed that GPx was clearly (*p* = 0.004) more intensive in the infertile group (6.559 nmol·min^−1^·mL^−1^) compared to the control (4.317 nmol·min^−1^·mL^−1^). The opposite situation was observed with GR, which was characterized by significantly (*p* = 0.043) higher activity in the healthy group (17.74 nmol·min^−1^·mL^−1^) compared with men with fertility disorders (15.97 nmol·min^−1^·mL^−1^). Differences among the activity of the rest of the enzymatic antioxidants (SOD and CAT) did not exhibit statistical significance (Table 1, Figure 1). The set of all output data is provided in Table S1 (Supplementary data) at the end of the paper.

### 3.2. Malondialdehyde Concentration, Enzymatic Antioxidants and Chemical Elements

The results of MDA concentrations were unexpected: more intensive lipid peroxidation (*p* = 0.031) was observed in the serum of the healthy control (20.94 µM) than in the infertile group (12 µM) (Table 1, Figure 1). An analysis of the correlations among enzymatic antioxidative mechanisms, MDA and chemical elements (Table 2) indicated that the most frequently correlating element in the healthy control group was boron. Applied to SOD and GPx, dependency was inversely proportional (*r* = −0.467 and −0.608 respectively). In the case of GR and MDA, correlation was positive (*r* = 0.407 and 0.326 respectively). Furthermore, GR correlated with silver, barium, aluminum and boron. GPx, as well as boron, correlated with arsenic. SOD showed interactions with sodium, lithium, barium (positive correlations) and boron (negative correlation) (Table 2). The correlativity of antioxidative parameters, MDA and chemical elements in the infertile group revealed important participation of boron, barium, aluminum, and tin, while the most relevant modulators of antioxidative defensive mechanisms in this group, beside boron (B-GPx: *r* = −0.587, B-GR: *r* = 0.590), appeared to be aluminum (Al-GR: *r* = −0.627) and barium (Ba-GR: *r* = −0.411). However, only boron appeared to be equally relevant in both analyzed groups: in the infertile group, it correlated with GPx (*r* = −0.587) and GR (*r* = 0.590), and in the healthy control group with SOD (*r* = −0.467), GPx (*r* = −0.608) and GR (*r* = 0.407) (Table 2). On the other hand, MDA showed only weak correlations with chemical elements, but these were evidently more pronounced in the infertile group, i.e., negative interactions with calcium (Ca-MDA: *r* = −0.393) and barium (Ba-MDA: *r* = −0.353) and positive with tin (Sn-MDA: *r* = 0.300) and boron (B-MDA: *r* = 0.338). In the control group, only boron demonstrated interactions with a marker of lipid peroxidation (B-MDA: *r* = 0.326) (Table 2). On the other hand, a relatively strong relationship between GR and GPx in the infertile group (*r* = −0.546), as well as weaker correlations in the healthy control group (SOD-GPx: *r* = 0.370, SOD-GR: *r* = −0.337), revealed mutual regulation of antioxidants and emphasized the inter-related nature of the entire system (Table 1).

### 3.3. Relationships with Polymorphism of Gene IL-4v.C589T (rs2243250)

**Figure 2:**
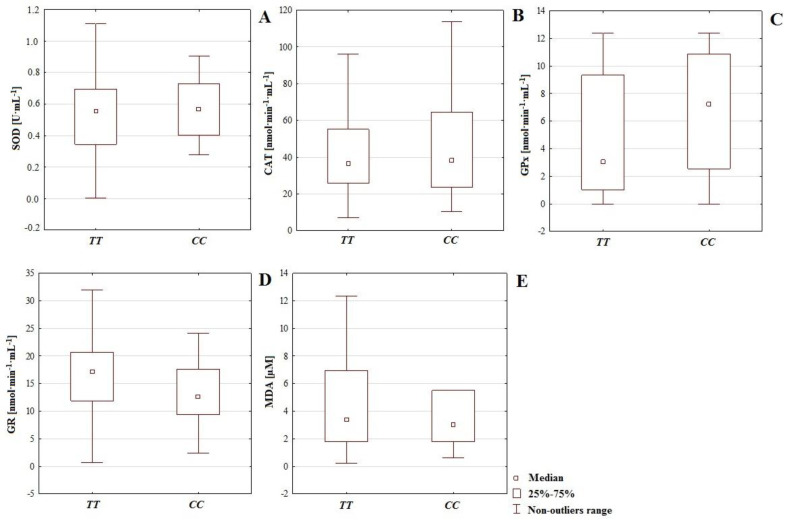
Activity of superoxide dismutase SOD (**A**), catalase CAT (**B**), glutathione peroxidase GPx (**C**), and glutathione reductase GR (**D**); concentration of malondialdehyde MDA (**E**) regarding *IL-4*v.*C589T* (rs2243250) genotypes *CC* and *TT*.

**Figure 3:**
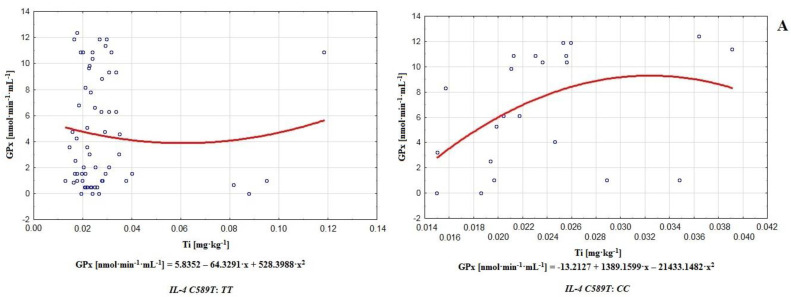

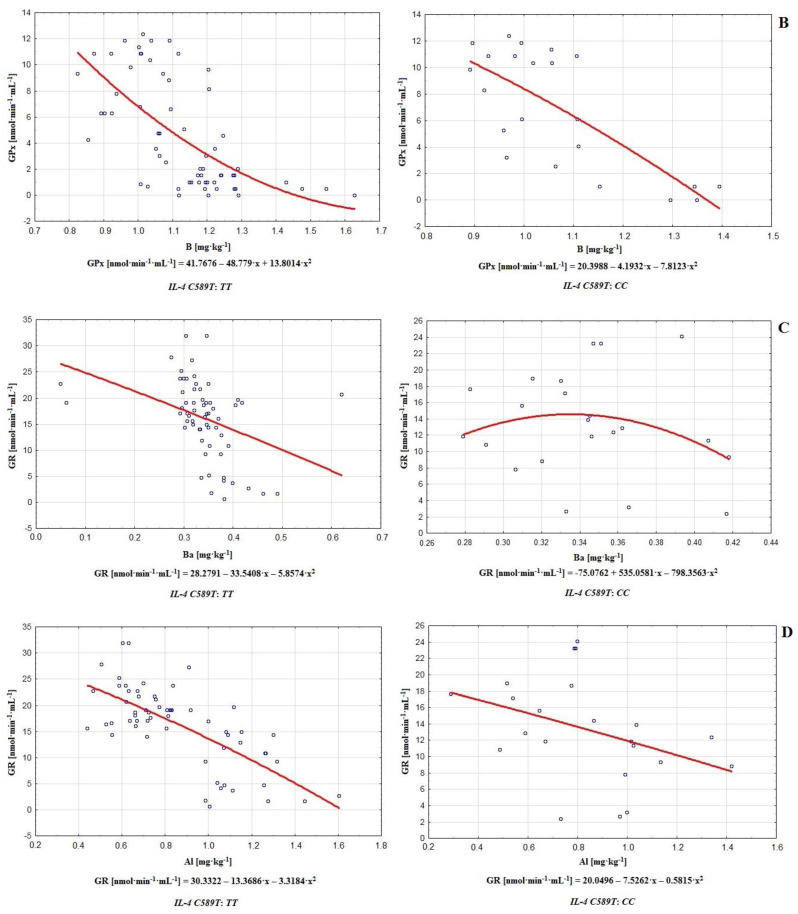

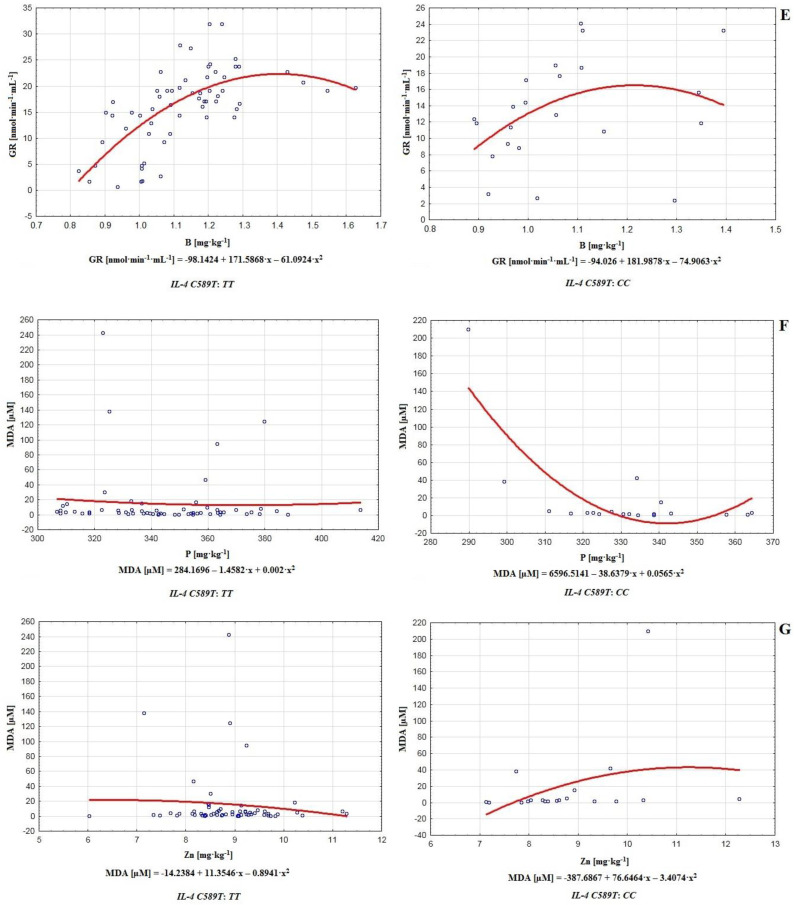
Correlations among glutathione peroxidase GPx and titanium Ti (**A**), glutathione peroxidase GPx and boron B (**B**), glutathione reductase GR and barium Ba (**C**), glutathione reductase GR and aluminum Al (**D**), glutathione reductase GR and boron B (**E**), malondialdehyde MDA and phosphorus P (**F**), malondialdehyde MDA and zinc Zn (**G**) regarding *IL-4*v.*C589T* (rs2243250) genotypes *CC* and *TT*.

In the infertile group, the polymorphism of gene *IL-4*v.*C589T* (rs2243250) was confirmed and 31 mutated homozygotes *TT* were noted (frequency of occurrence 0.72), along with 12 wild type homozygotes *CC* (frequency of occurrence 0.27). Heterozygotes *CT* were not detected. In the control group, this polymorphism was confirmed in 44 subjects. Among them, 34 mutated homozygotes *TT* (frequency of occurrence 0.77) and 10 wild type homozygotes *CC* (frequency of occurrence 0.22) were noted. In the control group, similar to the infertile group, heterozygotes *CT* were not noted. Electrophoretic section of the PCR product for gene *IL-4*v.*C589T* (rs2243250) resulted in one stripe at a height of 195pz. As the result of digestion with restrictive enzyme *AvaII*, stripes at various heights were observed. A single stripe at 195 bp indicated mutant type homozygote *TT*, while a single stripe at 177 bp indicated wild type homozygote *CC*. An analysis of the polymorphism of gene *IL-4*v.*C589T* (rs2243250) did not show significant difference regarding the occurrence of particular genotype in the two groups. Genotype *TT* was present in 72.09% of participants from the infertile group and 77.27% from the healthy control group. Genotype *CC* was present in 27.91% and 22.73%, respectively, of infertile and healthy participants (Table 3). Based on the frequency of these genotypes in the healthy and infertile groups, the direct impact of genetic factors on fertility could be excluded (lack of significant difference in frequency of genotypes; *p* = 0.578). However, for the polymorphism of gene *IL-4*v.*C589T* (rs2243250), it was possible to compare how genotypes shaped the status of antioxidative defenses, as well as correlations of enzymatic antioxidants with chemical elements and MDA. On this basis, it was possible to draw conclusions about the indirect influence of genetic factors on male reproductive potential (Table 3 and Table 4, Figure 2 and Figure 3). An analysis of enzymatic antioxidative activity and lipid peroxidation regarding genotypes (polymorphism of gene *IL-4*v.*C589T* (rs2243250) showed that none of the compared parameters exhibited significant difference between groups. In the case of GR, higher activity occurred in men with genotype *TT*, while for the rest of the parameters, higher values occurred in men with genotype *CC* (Table 3, Figure 2). Higher activity of SOD (0.570 U·mL^−1^), CAT (51.861 nmol·min^−1^·mL^−1^) and GPx (6.792 nmol·min^−1^·mL^−1^) in the group with genotype *CC* compared to men with genotype *TT* (0.561 U·mL^−1^, 45.823 nmol·min^−1^·mL^−1^ and 4.722 nmol·min^−1^·mL^−1^ respectively) may indicate that antioxidative defense, at the first line of defense, is more intense in the presence of genotype *CC*. On the other hand, a higher concentration of MDA in the group with genotype *CC* (18.094 µM) compared to *TT* (15.171 µM) is a symptom of more intense lipid peroxidation Table 3, Figure 2). An analysis of the correlations between enzymatic antioxidants and MDA in the case of men with genotypes *TT* or *CC* (polymorphism of gene *IL-4*v.*C589T* (rs2243250) revealed numerous interactions (Table 3, Figure 3). Positive and negative correlations between antioxidants provide information about their cooperation and mutual regulation, providing evidence of the cooperative character of the antioxidative system (genotype *TT*: GR-SOD *r* = −0.364, GR-GPx *r* = −0.500; *CC*: GPx-SOD *r* = 0.355, GPx-CAT *r* = −0.426). However, the group with genotype *CC* was distinguished by the occurrence of several correlations between antioxidants and MDA (CAT-MDA: *r* = 0.462; GPx-MDA: *r* = −0.479; GR-MDA: *r* = 0.304) that may have been indicators of possible more significant destabilization of antioxidative defenses in the group with genotype *CC* (compared to *TT*, where such interactions did not occur) (Table 3). Correlations of antioxidative mechanisms and MDA with concentrations of chemical elements in the group of men with genotype *TT* indicated important relationships in the case of GR-Ba (*r* = −0.549), GR-Al (*r* = −0.641) and GR-B (*r* = 0.675). Particularly significant appeared to be the negative correlation between GPx and B (*r* = −0.664), as well as the positive correlation between GR and B (*r* = 0.675). All other correlations were characterized by weaker power (*r* < 0.5); Table 4, Figure 3. An analogous analysis of the correlations between the activity of antioxidants, MDA, and chemical elements in the group of men with genotype *CC* revealed relatively strong relationships in the case of Na-SOD (*r* = 0.500), MDA-P (*r* = −0.518), MDA-Zn (*r* = 0.520), GPx-Ti (*r* = 0.548), and GPx-B (*r* = −0.632); Table 4, Figure 3. Finally, in the group with genotype *TT*, the strongest interactions were observed with Ba-GR (*r* = −0.549), Al-GR (*r* = −0.641), B-GPx (*r* = −0.664), and B-GR (*r* = 0.675). On the other hand, in the case of genotype *CC*, the strongest interactions occurred between Na-SOD (*r* = 0.500), P-MDA (*r* = −0.518), Zn-MDA (*r* = 0.520), Ti-GPx (*r* = 0.548), and B-GPx (*r* = −0.632); Table 4, Figure 3. Therefore, predominant participation in the modulation of enzymatic antioxidative mechanisms according to genotype was observed with Ba, B, Al, and Na. In a group with genotype *CC*, compared to *TT*, cadmium demonstrated the most significant role in terms of shaping antioxidative response and lipid peroxidation (Cd-CAT: *r* = −0.365, Cd-GPx: *r* = 0.494, Cd-MDA: *r* = −0.415). Additionally, in the group with genotype *CC*, relatively strong correlations were observed between phosphorus and MDA (*r* = −0.518), as well as between zinc and MDA (*r* = 0.520). The level of MDA in this group may have also been shaped by chemical elements such as boron (B-MDA; *r* = 0.428), barium (Ba-MDA; *r* = −0.465), and calcium (Ca-MDA; *r* = −0.447); Table 4, Figure 3.

## 4. Discussion

The process of differentiation of sperm during spermatogenesis is connected with a reduction in the content of cytoplasm. Therefore, sperm are characterized by relatively low intracellular antioxidant activity. On this level, the antioxidative system protecting against oxidative stress is constituted by SOD, nuclear GPx, thioredoxin (TRX), peroxiredoxin (PRDX), and thioredoxin reductase (TRD). Because of this, an even more important role in ROS counteraction is fulfilled by the enzymatic antioxidants present in seminal plasma, namely SOD, CAT, and GPx. A supporting function is realized by several nonenzymatic antioxidants also contained in seminal plasma, like ascorbic acid, α-tocopherol, uric acid, taurine, and hypotaurine [47]. Additionally, prostasomes (secreted by the prostate gland and organized in organelles) participate in communication between prostate cells and sperm, evoking a motility pattern in sperm cells that is essential for oocyte fertilization as well as being engaged in protection against oxidative stress. In this respect, prostasomes interact with neutrophils, decreasing their potential to generate superoxide anion radicals after stimulation [35]. A separate kind of unfavorable oxidative modification which mainly affects biological membranes is lipid peroxidation. ROS overproduction may trigger this phenomenon via nonenzymatic (iron-dependent) or enzymatic (catalyzed by lipoxygenase) mechanisms. The products of lipoperoxidation exhibit high biological activity and are able to provoke cell death signals, thereby inducing programmed cell death. Undoubtedly, a decisive factor is a balance between the generation and elimination of ROS. Therefore, in the conditions of limited lipoperoxidation, cell survival is promoted (the process itself stimulates the production of an “adaptive answer”, which manifests in the mobilization of antioxidative systems). In contrast, in toxic conditions (high levels of lipid peroxidation), processes of apoptosis and cellular necrosis are promoted [48]. In this paper, we demonstrated that, regarding male fertility abnormalities, the results of analyses of MDA concentrations may be irrelevant; in the serum of normozoospermic men, the average concentration was higher (20.94 µM) than in those with fertility impairments (12.00 µM, *p* = 0.031); see Table 1. Although MDA level may not be correlated with sperm DNA fragmentation and oxidation, suggesting that some fundamental parameters for sperm quality may remain independent of MDA [49], past studies have emphasized the connection between intensive lipid peroxidation, increased MDA level, worsening sperm quality, and general reproductive potential [32,33].

It seems interesting to compare the intensity of lipoperoxidation and the values of MDA concentrations obtained in our studies, in men with infertility and healthy controls, against the research results available in the literature. A few researchers [50] have suggested that increased MDA may be also interpreted as a kind of acclimation mechanism. In certain organisms, particularly if redox signaling regulation is undisturbed, MDA may stimulate regulatory genes or even participate in cellular protection under conditions of oxidative stress. Therefore, transient increases of MDA may serve as a defensive signaling factor that participates in the mobilization of antioxidative mechanisms to counteract ROS. These suggestions led to the consideration of temporal increases in MDA concentration as a protective mechanism, and not as an indicator of damage. This is an interpretation that may be true of the healthy controls analyzed in our work (Table 1). Furthermore, some crucial methodological aspects may be decisive in determining the relevance, or lack thereof, of the obtained results of MDA concentrations for particular analysis and study issues [50]. However, in cases of persistent environmental stress, certain stressors tended to cause increments in the level of MDA in dose-dependent manner. In such situations, the opposite effect may be noticeable regarding the activity of enzymatic antioxidants, which may undergo considerable reduction. For example, in experiments by Gutiérrez-Salinas et al. (2013) [51], sodium fluoride at concentrations of 0, 7, 28, 56 and 100 µg·mL^−1^ caused an evident increase of MDA in human erythrocytes accompanied by a reduction in the activity of SOD, CAT, and GPx during incubation with vitamin E (NaF 100 µg·mL^−1^ + various doses of vitamin E 1, 2.5, 5 and 10 µg·mL^−1^), partially restoring the activity of antioxidants as well as reversing the toxic effects caused by the applied stressor. A noticeable reduction of MDA was also observed [51].

Crucial for sperm quality are also interactions among chemical elements that are needed for certain biological functions. Cytosolic SOD utilizes both copper and zinc in dismutation of superoxide anion radicals, while cooperation between iron and sulfur is essential for the functionality of ferredoxin. The conversion of oxygen to water with cytochrome oxidase requires the presence of iron and copper [52]. Ions of sodium, potassium, and calcium, as well as the effective functionality of their transport channels, are fundamental for ensuring of sperm motility and correct maturation [53]. Therefore, the presence of sodium, potassium, and calcium is essential for the success of the process of reproduction in physiological conditions. In this paper, we demonstrate a correlation between Na concentration and SOD activity (infertile group: *r* = 0.309; control: *r* = 0.310; men with *CC* genotype regarding *IL-4*v.*C589T* (rs2243250) polymorphism: *r* = 0.500; Table 2 and Table 4), indicating a possible augmentation of antioxidative potential. Negative correlations were also observed between concentration of Ca and level of MDA in the infertile group (*r* = −0.393) and calcium, magnesium, and phosphorus regarding *IL-4*v.*C589T* (rs2243250) polymorphism (genotype *CC r* = −0.477, *r* = −0.326, and *r* = −0.518, respectively), indicating the importance of these macro-elements in terms of reducing lipid peroxidation (Table 2 and Table 4). Ca is considered to be beneficial for fertility, supporting sperm motility and enabling acrosome reactions to occur. On the other hand, while Mg is also necessary for sperm motility and effective spermatogenesis, it also influences intracellular Ca antagonist. This interaction confirms the necessity of cooperative actions among macro-elements for the preservation of male fertility [54]. Additionally, microelements play crucial roles in sperm quality; the most important are Zn, which improves sperm motility and is a cofactor of essential enzymatic reactions, Fe and Cu, which are similarly engaged in redox processes and antioxidant metabolism through their activity as cofactors, and Se, which is a component of glutathione peroxidase–selenoproteins present in sperm midpiece [54]. A negative correlation between barium concentration and activity of GR (*r* = −0.411 in infertile group and *r* = −369 in control), and a positive correlation between Ba and SOD in the infertile and control groups (*r* = 0.329 and *r* = 0.311) were noted (Table 2).

Recent studies have considered barium as a toxic contaminant which induces ROS formation, thereby degrading proteins, nucleic acid, lipids, and cellular antioxidants. The accumulation of relatively high doses of barium can also stimulate activity of certain antioxidants, e.g., catalase [55]. Additionally, barium is commonly present in drinking water, and its soluble salts are generally more toxic than its insoluble ones. The negative effect of Ba is based on blocking the K^+^ and Na-K^+^ channels in the cellular membrane, which destabilizes potassium transfer. Extreme manifestations result in the paralysis of skeletal muscles [56]. Therefore, this chemical element exerts an ambiguous influence on antioxidative defenses, stimulating the first line of defense while weakening the second.

Another chemical element with toxic potential creating a multi-faceted threat to health is aluminum. In this paper, we noted negative correlations in the infertile group and control between concentration of Al and GR activity (*r* = −0.627 and *r* = −0.340 respectively; see Table 2). Although aluminum is mainly considered as a potent neurotoxic factor, it is able to perturb redox balance, increasing lipid peroxidation and decreasing antioxidant enzyme activity. The effect of such a reduction may be observed regarding CAT, glutathione-S-transferase, GPx or, as noted in this paper, GR. On the other hand, Al may affect SOD in an ambiguous way, causing a decrease or increment in enzyme activity, with the latter resulting in hydrogen peroxide overburden [57]. The negative effects of Al on semen quality and general reproductive condition are well studied in human and animal models, and involve increased oxidative stress, intensification of inflammatory processes, reduced sperm production, decreased sperm motility and count, increment in defective sperm numbers, reductions in weight of epididymis and testes, and reduced serum testosterone and luteinizing hormone production. Simultaneously, aluminum influences the levels of important chemical elements in the testis, causing an increase in iron and zinc and a decrease of copper, which may be detrimental to antioxidative defenses. Oligospermic men generally show higher Al concentrations compared to healthy individuals [58]. This confirms the generally unfavorable impact of this chemical element on sperm antioxidative defenses.

Boron also may be considered a potential threat. It exerts ambiguous but cumulative effects on the activity of certain enzymatic antioxidants like SOD, CAT, and GR [59]. Meanwhile, animal research has also shown that boron increases the activity of SOD erythrocyte as well as the levels of CAT and GPx in blood and cells. It may even exert an anti-inflammatory effect, scavenging for leukocytes and reducing the impact of oxidative bursts. Regarding sex hormones, B supplementation can cause an increase in free testosterone, a decrease in estradiol and even a reduction of certain inflammatory biomarkers like interleukin 6, tumor necrosis factor, or high-sensitivity C-reactive protein. This effect prompt us to consider boron as an androgen amplifier [60,61]. The concentration of this element correlated negatively with GPx activity in the infertile and control groups (*r* = −0.587 and *r* = −0.608, respectively). Simultaneously, boron was positively correlated with GR in both groups (*r* = 0.590 and *r* = 0.407, respectively; see Table 2), which suggests a stimulating impact on the second line of defense by a restrictive effect on the first one. Animal studies have confirmed the negative impact of boric acid and borates on the reproductive system, although these results may not be relevant to male human fertility, because the experimental doses in animal models were probably significantly highed than those that could result from typical occupational exposure [62,63]. In this work, both in the case of men with fertility disorders and healthy controls, a positive correlation between B and MDA was observed (*r* = 0.338 and *r* = 0.326 respectively), which may imply that boron contributes to the stimulation of lipid peroxidation (Table 2).

In this paper, we tried to reveal relationships between the polymorphism of gene *IL−4* (chromosome 5) and male infertility in the population of central Poland. In the case of the polymorphism of *IL-4*v.*C589T* (rs2243250), we proved the non-existence of statistically significant differences between the infertile and control groups regarding the frequency of genotypes (chi^2^ = 0.309, *p* = 0.578, Table 3). On this basis, we proved the non-existence of direct relationships between the studied polymorphism and fertility disorders in the analyzed population. However, it is worth considering the possibility of an indirect impact of this polymorphism on male reproductive potential. In this respect, our analysis of the polymorphism of *IL-4*v.*C589T* (rs2243250) allowed us to identify some important differences connected with the presence of genotypes *TT* and *CC* in the studied men (see Table 3 and Table 4; Figure 2 and Figure 3). These genotypes were found to mediate the relationships among chemical elements and antioxidants. Certain chemical elements are directly connected with oxidative defense or semen quality. Macro-elements, e.g., calcium and phosphorus, are fundamental for sperm functional integrity; magnesium maintains the activity of important seminal enzymes, and its deficiency increases the risk of premature ejaculation [64]. Crucial microelements including zinc, copper, iron, nickel, and manganese are fundamental cofactors for the functionality of antioxidative system components. Zinc is also engaged in proper capacitation. Finally, toxic heavy metals (Cd, Hg) constitute a threat both antioxidative balance and general reproductive potential, while their negative influence on sperm quality reflects a link between environmental pollution and decreasing fertility [64].

The results of this paper suggest that in addition to the aforementioned elements, Mo, Li, V, Co, Ag, Ti, Tl, Sr, Sn, and Be may influence the efficiency of sperm antioxidative defenses or lipid peroxidation processes (genotype *CC* of gene *IL-4*v.*C589T* (rs2243250) polymorphism; Mo-CAT *r* = 0.329, Li-SOD *r* = 0.316, V-SOD *r* = 0.351, Co-GPx *r* = 0.358, Ag-CAT *r* = −0.313, Ti-CAT *r* = −0.311, Ti-GPx *r* = 0.548, Ti-GR *r* = 0.393, Tl-SOD *r* = 0.471, Tl-GPx *r* = 0.385, Sr-SOD *r* = 0.304, Sn-GR *r* = 0.331, and Be-SOD *r* = −0.380; see Table 4). It was also apparent that the number of correlations in the case of the *CC* genotype considerably surpassed those with *TT*. This observation revealed a magnitude of possible modulators, and may indicate greater destabilization of antioxidative potential in the case of genotype *CC* compared to *TT* (polymorphism of gene *IL-4*v.*C589T* (rs2243250) (Table 4). In the matter of particular antioxidative mechanisms in the groups of men with genotypes *TT* or *CC* (polymorphism of gene *IL-4*v.*C589T* (rs2243250), both positive and negative correlations among antioxidants were noted. The actions of antioxidants in terms of counteracting ROS generation or capturing generated radicals somehow require their cooperation in either of the main lines of defense. Furthermore, antioxidative enzymes are able to catalyze the regeneration or synthesis of non-enzymatic antioxidants, which reveals the cooperative nature of the entire system [65]. Independently, in the group with genotype *CC*, several correlations with MDA were observed: CAT-MDA (*r* = 0.462), GPx-MDA (*r* = −0.479), and GR-MDA (*r* = 0.304) (Table 3). It is well established that enzymatic antioxidants alone or in combination generally improve sperm parameters, i.e., CAT by increasing sperm motility and decreasing abnormal spermatozoa; GPx in combination with CAT and SOD by extending sperm viability, augmenting motility, and reducing DNA fragmentation; and finally SOD alone by improving motility and viability [66]. Dysfunctional and poorly motile sperm are characterized by increased unsaturated fatty acid content, making them sensitive to oxidative damage. Furthermore, incorrect sperm morphology also favors lipid peroxidation. Finally, dead spermatozoa are potent producers of ROS and inducers of DNA fragmentation in correct gametes [67]. Therefore, the connection between MDA concentration and the activity of antioxidants regarding sperm quality is irrefutable. These relationships may also bring about more significant destabilization of antioxidative defenses in the group with genotype *CC* (comparing to *TT*).

Relationships between genotype *TT* or *CC* (*IL-4*v.*C589T* (rs2243250) polymorphism) and interactions among chemical elements and antioxidants were demonstrated, with the most significant ones in the case of *TT* being Ba-GR (*r* = −0.549), Al-GR (*r* = −0.641), B-GPx (*r* = −0.664), B-GR (*r* = 0.675) (Table 4, Figure 3). In fact, certain widely used barium compounds are able to increase antioxidative system response, e.g., barium oxide nanoparticles tend to increase catalase and superoxide dismutase levels while lowering glutathione activity. Long-term exposure to barium oxide also triggers DNA damage, implying its potential to increase ROS generation, augment lipid peroxidation, and initiate genotoxic effects [68]. B and Al significantly influence antioxidative efficiency, improving or decreasing the activity of certain antioxidants. For example, AlCl_3_ is able to induce lipid peroxidation and may even augment catalase activity [69], while boron stimulates or exerts an ambiguous effect on SOD, CAT, and GPx [60]. Therefore the strongest effects on the functionality of antioxidative defenses in the group with genotype *TT* were observed for barium, boron, and aluminum. On the other hand, among men with genotype *CC*, the strongest correlations occurred between Ti-GPx (*r* = 0.548) and B-GPx (*r* = −0.632); see Table 4. Additionally, it is worth noting that on this level, aluminum and boron correlated very frequently with antioxidants (Al-SOD *r* = 0.353, Al-GPx *r* = 0.436, Al-GR *r* = −0.390, B-SOD *r* = −0.364, B-GPx *r* = −0.632, and B-GR *r* = 0.392; see Table 4), acting as important modulators of the antioxidative barrier. In contrast to the group with genotype *TT*, in the case of *CC*, relatively strong correlations between phosphorus and MDA (*r* = −0.518) and between zinc and MDA (*r* = 0.520) were noted (Table 4, Figure 3).

Notwithstanding the generally beneficial roles of these chemical elements in physiological concentrations, there are some unfavorable effects when they are in excess or deficiency, e.g., phosphorus excess may increase the risk of erectile dysfunction, low testosterone production, abnormal sperm functionality, as well as the induction of inflammation, apoptosis and oxidative stress, which implies a link between phosphorus level and intensity of lipid peroxidation [70]. In contrast, zinc deficiency may be connected with diminished sperm count, increased oxidative damage, higher MDA level, lower SOD activity, and even impotence [71]. On the other hand, Zn overload may be cytotoxic, leading to cell death. Additionally, in such cases, oxidative stress is a key factor connected with increased levels of NADPH oxidase [72]. The ambiguity of the interactions of phosphorus and zinc with MDA may be evidence of stronger destabilization of antioxidative defenses in the group with genotype *CC*. The significantly positive Zn-MDA correlation was alarming because, according to the data, zinc is essential for the progression of spermatogenesis, proper sperm motility, maintenance of germ cells, DNA repair, ROS scavenging, and antioxidative efficiency [71]. As a chemical element with antioxidative proprieties, it should participate in the reduction of ROS levels and downgrade MDA, as well as increasing the potential of antioxidants [73].

Regarding the impact of genotype *CC* or *TT* (polymorphism of gene *IL-4*v.*C589T* (rs2243250) on the activity of antioxidative mechanisms and MDA level, slightly higher concentrations of MDA (more intense lipoperoxidation) accompanied genotype *CC* (18.094 µM), compared to *TT* (15.171 µM). However, genotype *TT* is connected with increased GR activity (15.966 nmol·min^−1^·mL^−1^, *CC*: 13.306 nmol·min^−1^·mL^−1^). On the other hand, the group with genotype *CC* was distinguished by higher activity of SOD (0.570 U·mL^−1^, *TT*: 0.561 U·mL^−1^), CAT (51.861 nmol·min^−1^·mL^−1^, *TT*: 45.823 nmol·min^−1^·mL^−1^), and GPx (6.792 nmol·min^−1^·mL^−1^, *TT*: 4.722 nmol·min^−1^·mL^−1^); (Table 3). These results suggest a more effective functionality of the first or second lines of defense according to the presence of the *CC* or *TT* genotypes. They also confirm that genetic factors shape chemical element economy and antioxidative potential. The functionality of antioxidative defenses requires the cooperation of many mechanisms, and may be shaped by chemical elements economy or genetic factors, which also indicates the multifactorial nature of male infertility. Therefore, cooperation among sperm enzymatic antioxidants is necessary for effective antioxidation [10,74]. In our study, we noted significant differences in the activities of antioxidative enzymes in both groups, i.e., GPx (*p* = 0.004) and GR (*p* = 0.043). The activity of GPx was higher in the infertile group (6.559 nmol·min^−1^·mL^−1^) compared to the control (4.317 nmol·min^−1^·mL^−1^), while the activity of GR was lower in men with fertility disorders (15.97 nmol·min^−1^·mL^−1^) compared to controls (17.74 nmol·min^−1^·mL^−1^); (Table 1, Figure 1). This revealed significant differences in the functionality of the first and (partly) second lines of defense between groups. Furthermore, in the infertile group, we noted a negative correlation between GR and GPx (*r* = −0.546) which suggests mutual regulation among enzymatic antioxidants (Table 1).

In summary, chemical element economy shapes male reproductive condition, while the functionality of enzymatic antioxidants differs between men with fertility disorders and healthy controls. The occurrence of correlations among antioxidants emphasizes the cooperative character of the antioxidative barrier of sperm against oxidative stress. The analysis of MDA levels may be irrelevant in the matter of male reproductive potential (the higher MDA concentration in the serum of healthy controls compared to the infertile group appears controversial). The polymorphism of gene *IL-4*v.*C589T* (rs2243250) was not directly connected with male reproductive condition in the analyzed population. However, men with genotype *TT* and *CC* demonstrated diverse models of interaction between economy of chemical elements and enzymatic antioxidant system activity. Correlations among chemical elements, enzymatic antioxidants, and MDA were more numerous in the case of genotype *CC* compared to *TT*. Genotype *TT* is connected with higher enzymatic antioxidant activity, i.e., the second line of defense (GR), while in the case of genotype *CC*, the first line of defense is characterized by a higher intensity (SOD, CAT, GPx). Nevertheless, correlations among antioxidants indicated their cooperative nature and mutual regulation (Table 3, Figure 2). The economy of chemical elements is important for enzymatic antioxidative defense. In the matter of the polymorphism of *IL-4*v.*C589T* (rs2243250), genotype may also be a modulating factor. Boron, barium, and aluminum may be considered the most significant modulators of enzymatic antioxidative potential (Table 2 and Table 4, Figure 3). Additionally, in the group with genotype *CC*, a significant influence on antioxidative defense and lipid peroxidation was found to be exerted by cadmium (Table 4). The results of this paper should heighten concerns about the status of the natural environment and lifestyles. Oxidative stress is a consequence of an accumulation of negative factors that decrease male reproductive potential. Antioxidative defense, economy of chemical elements, environmental factors, and genetic aspects are all interconnected and jointly shape male reproductive condition. Enzymatic antioxidants also undergo mutual regulation, as confirmed by the observed correlations among them. The polymorphism of *IL-4*v.*C589T* (rs2243250) indirectly influences enzymatic antioxidative defense and interactions among antioxidants and chemical elements (Table 3 and Table 4, Figure 2 and Figure 3). The obtained results may be helpful in diagnoses of male infertility (reduction of idiopathic cases, more effective treatment), reducing the number of idiopathic cases, and the creation of more effective treatments, while the identification of relationships among environmental stressors and fertility disorders may help to eliminate or reduce the impact of factors which are detrimental to fertility, as well as the perceived importance of immunogenetic factors in terms of shaping the defensive abilities of an organism against damage to reproductive material. Based on our results, conclusions could be drawn about the role of oxidative stress in the induction of genetic polymorphisms which threaten male infertility. Subsequently, our findings may draw attention to factors which are harmful for male fertility (environmental and immunogenetic) and methods to minimize them.

## 5. Conclusions

1. The results of this study suggest more effective functionality of enzymatic antioxidants as the first line of defense (GPx) in men with fertility disorders, and a more intense second line of defense (GR) in men with normozoospermia.

2. Male fertility and antioxidative potential are shaped by many factors that cooperate and undergo mutual regulation for effective functionality of the overall defense mechanism against oxidative stress. This was confirmed by correlations on various levels: among antioxidants: GR-GPx *r* = −0.546 in the infertile men; among chemical elements and antioxidants: B-SOD *r* = −0.467, B-GPx *r* = −0.608, B-GR *r* = 0.407 in healthy controls; and B-GPx *r* = −0.587, B-GR *r* = 0.590, Al-GR *r* = −0.627 in the infertile group. Finally, also regarding genotypes (polymorphism of gene *IL-4*v.*C589T* (rs2243250), correlations appear among antioxidants: GPx-GR *r* = −0.500 in men with genotype *TT*; GPx-CAT *r* = −0.426 in men with genotype *CC* and among chemical elements and antioxidants: Ba-GR *r* = −0.549, Al-GR *r* = −0.641, B-GPx *r* = −0.664, and B-GR *r* = 0.675 in men with genotype *TT*; Al-GPx *r* = 0.436 and B-GPx *r* = −0.632 in men with genotype *CC*.

3. The functionality of antioxidative mechanisms depends on interactions with chemical elements (B, Ba, Ca, Na, and Al). Macro-elements may participate in the reduction of lipid peroxidation, conditioning sperm maturation (Ca, P) or stimulating the activity of antioxidants (Na-SOD). Enzymatic antioxidants, lipid peroxidation, and economy of chemical elements are connected with male fertility.

4. The frequency of genotypes in *IL-4*v.*C589T* (rs2243250) revealed no difference between the infertile and control groups. Therefore, this polymorphism does not exert a direct influence on fertility in the studied population.

5. Genotype *TT* or *CC* in *IL-4*v.*C589T* (rs2243250) indirectly shaped interactions between antioxidants, chemical elements, and lipid peroxidation (more correlations in the group with genotype *CC*).

6. Genotype *TT* or *CC* in *IL-4*v.*C589T* (rs2243250) does not significantly influence enzymatic antioxidative mechanisms or overproduction of MDA. However, a higher activity of MDA in the case of *CC* compared to *TT* indicated that the former may predispose individuals to more intense lipoperoxidation as well as larger destabilization of antioxidative defenses. Correlations among antioxidants, chemical elements, and MDA in men with genotype *CC* allowed us to draw conclusions about more significant destabilization of antioxidative barriers in this group. The polymorphism of *IL-4*v.*C589T* (rs2243250) did not exert a decisive effect on male fertility in the studied groups. However, it was shown to influence interactions among antioxidants, chemical element economy, and lipid peroxidation, thereby indirectly shaping antioxidative potential.

7. This is the first study to apply such an approach to research on the dependencies analyzed herein; therefore, this research is innovative in nature.

## Figures and Tables

**Table 1:** Activity of superoxide dismutase SOD, catalase CAT, glutathione peroxidase GPx, and glutathione reductase GR; concentration of malondialdehyde MDA and mutual interactions in healthy control group and men with infertility. Statistically significant differences and correlations are shown in bold.

U-Mann-Whitney Test																							
Parameter		Healthy										Infertile										*p*	
		$\bar{x}$	Me	Q1		Q3		min		max		$\bar{x}$	Me		Q1		Q3		min	max			
SOD [U·mL^−1^]		0.461	0.436	0.263		0.665		0.002		1.242		0.538	0.553		0.323		0.706		0.005	1.788		0.169	
CAT [nmol·min^−1^·mL^−1^]		45.69	39.93	23.52		55.15		2.462		157.074		44.59	34.58		23.81		48.43		3.561	176.932		0.450	
GPx [nmol·min^−1^·mL^−1^]		4.317	3.056	1.019		6.793		0.000		12.4		6.559	6.198		2.038		10.36		0.000	22.24		**0.004**	
GR [nmol·min^−1^·mL^−1^]		17.74	18.68	12.90		21.73		0.170		51.62		15.97	15.62		9.848		19.19		1.868	99.50		**0.043**	
MDA [µM]		20.94	5.214	2.357		15.23		0.214		242.6		12.00	2.917		1.713		6.214		0.214	209.7		**0.031**	
**Spearman correlation test**																							
Healthy											Infertile												
Parameter	SOD [U·mL^−1^]		CAT [nmol·min^−1^·mL^−1^]		GPx [nmol·min^−1^·mL^−1^]		GR [nmol·min^−1^·mL^−1^]		MDA [µM]		parameter			SOD [U·mL^−1^]		CAT [nmol·min^−1^·mL^−1^]		GPx [nmol·min^−1^·mL^−1^]			GR [nmol·min^−1^·mL^−1^]		MDA [µM]
SOD [U·mL^−1^]	-										SOD [U·mL^−1^]			-									
CAT [nmol·min^−1^·mL^−1^]	−0.233		-								CAT [nmol·min^−1^·mL^−1^]			−0.149		-							
GPx [nmol·min^−1^·mL^−1^]	**0.370**		−0.146		-						GPx [nmol·min^−1^·mL^−1^]			0.164		−0.143		-					
GR [nmol·min^−1^·mL^−1^]	**−0.337**		−0.070		−0.245		-				GR [nmol·min^−1^·mL^−1^]			−0.273		0.115		**−0.546**			-		
MDA [µM]	−0.232		0.045		−0.259		**0.321**		-		MDA [µM]			**−0.303**		0.164		−0.282			0.206		-

**Table 2:** Correlations between superoxide dismutase SOD, catalase CAT, glutathione peroxidase GPx, glutathione reductase GR, concentration of malondialdehyde MDA and Ca, Na, P, Li, Ag, Ba, Al, Sn, B, and As in healthy controls and men with fertility disorders. Statistically significant relationships are shown in bold.

Spearman Correlation Test											
Healthy						Infertile					
Element	SOD [U·mL^−1^]	CAT [nmol·min^−1^·mL^−1^]	GPx [nmol·min^−1^·mL^−1^]	GR [nmol·min^−1^·mL^−1^]	MDA [µM]	Element	SOD [U·mL^−1^]	CAT [nmol·min^−1^·mL^−1^]	GPx [nmol·min^−1^·mL^−1^]	GR [nmol·min^−1^·mL^−1^]	MDA [µM]
Ca [mg·kg^−1^]	0.068	0.054	0.261	−0.174	−0.160	Ca [mg·kg^−1^]	0.192	−0.005	0.203	−0.291	**−0.393**
Na [mg·kg^−1^]	**0.310**	−0.059	0.287	−0.214	−0.111	Na [mg·kg^−1^]	**0.309**	−0.121	0.095	−0.095	0.064
P [mg·kg^−1^]	−0.158	0.150	−0.218	0.144	−0.157	P [mg·kg^−1^]	**−0.315**	0.080	0.162	0.035	−0.064
Li [mg·kg^−1^]	**0.301**	−0.152	0.203	−0.039	−0.068	Li [mg·kg^−1^]	−0.076	−0.223	0.172	−0.132	0.032
Ag [mg·kg^−1^]	−0.079	−0.237	−0.146	**0.311**	0.055	Ag [mg·kg^−1^]	0.008	−0.171	0.001	0.285	0.072
Ba [mg·kg^−1^]	**0.311**	−0.010	0.290	**−0.369**	−0.186	Ba [mg·kg^−1^]	**0.329**	0.023	0.194	**−0.411**	**−0.353**
Al [mg·kg^−1^]	0.099	0.131	0.145	**−0.340**	0.017	Al [mg·kg^−1^]	0.169	−0.113	**0.360**	**−0.627**	−0.235
Sn [mg·kg^−1^]	−0.057	−0.071	−0.041	0.246	−0.041	Sn [mg·kg^−1^]	−0.151	−0.063	−0.206	**0.401**	**0.300**
B [mg·kg^−1^]	**−0.467**	0.128	**−0.608**	**0.407**	**0.326**	B [mg·kg^−1^]	−0.156	0.091	**−0.587**	**0.590**	**0.338**
As [mg·kg^−1^]	−0.118	0.014	**−0.304**	0.179	0.170	As [mg·kg^−1^]	−0.084	0.275	−0.268	0.140	0.146

**Table 3:** Frequency of genotypes (*IL-4* v.*C589T* (rs2243250) in men with fertility disorders and healthy controls, as well as activity of superoxide dismutase SOD, catalase CAT, glutathione peroxidase GPx, glutathione reductase GR, concentration of malondialdehyde MDA and mutual interactions regarding *IL-4-*v.*C589T* (rs2243250) genotypes *CC* and *TT*. Statistically significant differences and correlations are shown in bold.

Frequency of Genotypes (Chi^2^ Pearson Test)																								
Group			Polymorphism of Gene *IL-4*v.*C589T* (rs2243250)																	χ2			*p*	
			Genotype *TT*									Genotype *CC*												
Infertile		n	31									12								0.309			0.578	
		%	72.09									27.91												
Healthy		n	34									10												
		%	77.27									22.73												
**U-Mann-Whitney test**																								
Parameter		Genotype *TT*										Genotype *CC*										*p*		
		$\bar{x}$	Me	Q1		Q3		Min		Max		$\bar{x}$	Me		Q1		Q3		Min	Max				
SOD [U·mL^−1^]		0.561	0.555	0.343		0.696		0.002		1.788		0.570	0.569		0.402		0.728		0.276	0.904		0.604		
CAT [nmol·min^−1^·mL^−1^]		45.823	36.699	26.018		55.147		7.078		145.950		51.861	38.267		23.515		64.326		10.357	176.932		0.947		
GPx [nmol·min^−1^·mL^−1^]		4.722	3.056	1.019		9.340		0.000		12.396		6.792	7.217		2.547		10.868		0.000	12.396		0.078		
GR [nmol·min^−1^·mL^−1^]		15.966	17.149	11.886		20.715		0.679		31.921		13.306	12.650		9.339		17.659		2.377	24.111		0.074		
MDA [µM]		15.171	3.380	1.806		6.929		0.214		242.643		18.094	3.009		1.806		5.500		0.602	209.786		0.587		
**Spearman correlation test**																								
Genotype *TT*											Genotype *CC*													
Parameter	SOD [U·mL^−1^]		CAT [nmol·min^−1^·mL^−1^]		GPx [nmol·min^−1^·mL^−1^]		GR [nmol·min^−1^·mL^−1^]		MDA [µM]		parameter			SOD [U·mL^−1^]		CAT [nmol·min^−1^·mL^−1^]		GPx [nmol·min^−1^·mL^−1^]			GR [nmol·min^−1^·mL^−1^]			MDA [µM]
SOD [U·mL^−1^]	-										SOD [U·mL^−1^]			-										
CAT [nmol·min^−1^·mL^−1^]	−0.019		-								CAT [nmol·min^−1^·mL^−1^]			−0.045		-								
GPx [nmol·min^−1^·mL^−1^]	0.271		0.005		-						GPx [nmol·min^−1^·mL^−1^]			**0.355**		**−0.426**		-						
GR [nmol·min^−1^·mL^−1^]	**−0.364**		0.046		**−0.500**		-				GR [nmol·min^−1^·mL^−1^]			−0.012		−0.112		0.073			-			
MDA [µM]	−0.163		−0.059		−0.172		0.192		-		MDA [µM]			−0.028		**0.462**		**−0.479**			**0.304**			-

**Table 4:** Correlations among superoxide dismutase SOD, catalase CAT, glutathione peroxidase GPx, glutathione reductase GR, concentration of malondialdehyde MDA and Ca, Mg, Na, P, Mn, Fe, Zn, Mo, Li, V, Co, Ag, Ba, Ti, Tl, Sr, Al, Ni, Sn, B, Hg, Cd, and Be regarding *IL-4*v.*C589T* (rs2243250) genotypes CC and TT. Statistically significant relationships are shown in bold.

Spearman Correlation Test											
Genotype *TT*						Genotype *CC*					
Element	SOD [U·mL^−1^]	CAT [nmol·min^−1^·mL^−1^]	GPx [nmol·min^−1^·mL^−1^]	GR [nmol·min^−1^·mL^−1^]	MDA [µM]	Element	SOD [U·mL^−1^]	CAT [nmol·min^−1^·mL^−1^]	GPx [nmol·min^−1^·mL^−1^]	GR [nmol·min^−1^·mL^−1^]	MDA [µM]
Ca [mg·kg^−1^]	0.019	0.075	0.266	**−0.377**	−0.156	Ca [mg·kg^−1^]	0.342	0.049	0.129	−0.285	**−0.447**
Mg [mg·kg^−1^]	−0.099	0.066	−0.027	−0.060	−0.092	Mg [mg·kg^−1^]	−0.263	0.015	0.148	−0.241	**−0.326**
Na [mg·kg^−1^]	0.242	−0.081	0.197	−0.242	0.155	Na [mg·kg^−1^]	**0.500**	−0.027	0.203	0.128	−0.253
P [mg·kg^−1^]	−0.215	0.226	−0.154	0.125	−0.140	P [mg·kg^−1^]	−0.150	−0.131	0.026	−0.241	**−0.518**
Mn [mg·kg^−1^]	0.218	−0.169	0.263	−0.261	−0.024	Mn [mg·kg^−1^]	0.248	**0.350**	−0.288	−0.073	0.066
Fe [mg·kg^−1^]	−0.128	0.083	−0.074	0.006	−0.245	Fe [mg·kg^−1^]	0.207	−0.136	**0.337**	−0.244	−0.290
Zn [mg·kg^−1^]	−0.044	0.144	−0.193	−0.003	0.026	Zn [mg·kg^−1^]	0.137	**0.353**	−0.188	0.144	**0.520**
Mo [mg*kg^−1^]	−0.174	0.135	−0.127	0.104	0.084	Mo [mg·kg^−1^]	−0.049	**0.329**	−0.167	−0.191	0.265
Li [mg·kg^−1^]	0.043	0.003	0.090	0.012	0.050	Li [mg·kg^−1^]	**0.316**	0.098	0.211	0.115	−0.161
V [mg·kg^−1^]	0.035	−0.104	−0.175	0.232	0.022	V [mg·kg^−1^]	**0.351**	−0.256	0.212	0.004	−0.170
Co [mg·kg^−1^]	−0.024	0.067	0.065	−0.163	−0.193	Co [mg·kg^−1^]	0.293	0.011	**0.358**	0.085	0.088
Ag [mg·kg^−1^]	0.063	−0.296	−0.005	0.280	0.138	Ag [mg·kg^−1^]	0.200	**−0.313**	−0,088	−0.076	−0.257
Ba [mg·kg^−1^]	0.238	0.026	0.246	**−0.549**	−0.161	Ba [mg·kg^−1^]	0.100	−0.031	0.053	−0,116	**−0.465**
Ti [mg·kg^−1^]	0.013	0.192	0.038	0.114	0.011	Ti [mg·kg^−1^]	0.123	**−0.311**	**0.548**	**0.393**	−0.002
Tl [mg·kg^−1^]	−0.063	−0.076	0.023	−0.120	−0.077	Tl [mg·kg^−1^]	**0.471**	0.074	**0.385**	−0.273	−0,147
Sr [mg·kg^−1^]	0.185	0.124	−0.156	−0.005	0.101	Sr [mg·kg^−1^]	**0.304**	−0.124	−0.091	0.264	−0.245
Al [mg·kg^−1^]	0.149	0.039	**0.383**	**−0.641**	−0.168	Al [mg·kg^−1^]	**0.353**	−0.069	**0.436**	**−0.390**	**−0.365**
Ni [mg·kg^−1^]	0.082	−0.238	−0.064	−0.079	0.064	Ni [mg·kg^−1^]	0.082	0.262	−0.095	**−0.469**	0.022
Sn [mg·kg^−1^]	−0.136	−0.154	−0.228	**0.407**	0.104	Sn [mg·kg^−1^]	−0.133	−0.001	−0.111	**0.331**	0.112
B [mg·kg^−1^]	−0.137	0.006	**−0.664**	**0.675**	0.208	B [mg·kg^−1^]	**−0.364**	0.254	**−0.632**	**0.392**	**0.428**
Hg [mg·kg^−1^]	0.134	0.123	−0.027	−0.030	**−0.343**	Hg [mg·kg^−1^]	0.074	0.205	−0.286	−0.214	0.115
Cd [mg·kg^−1^]	0.033	0.169	−0.113	0.052	−0.099	Cd [mg·kg^−1^]	0.127	**−0.365**	**0.494**	−0.109	**−0.415**
Be [mg·kg^−1^]	0.048	−0.138	−0.085	−0.063	−0.040	Be [mg·kg^−1^]	**−0.380**	−0.062	−0.088	0.227	−0.086

## Data Availability

Data is contained within this article and the supplementary file.

## Supplementary Materials

The following are available online at https://www.mdpi.com/article/10.3390/antiox11050817/s1, Table S1: Statistically significant results of seminological studies, molecular analyzes of the polymorphisms of the genes located on chromosome 5 (*IL-4*v.*C589T* (rs2243250), activity of antioxidant enzymes (superoxide dismutase SOD, catalase CAT, glutathione peroxidase GPx, and glutathione reductase GR), concentration chemical elements (Ca, Mg, Na, P, Mn, Fe, Zn, Mo, Li, V, Co, Ag, Ba, Ti, Tl, Sr, Al, Sn, Ni, B, Hg, Cd, As, Be) and the intensity of lipoperoxidation (concentration of malondialdehyde MDA) in healthy controls and men with fertility disorders.
