# Supplementary material for: Enzymatic Antioxidant Defense and Polymorphic Changes in Male Infertility
Source: Antioxidants (Basel). 2022 Apr 22;11(5):817. doi: 10.3390/antiox11050817 (PMC9138092; doi:10.3390/antiox11050817)
Supplement: Supplementary file 1 [file antioxidants-11-00817-s001.zip › antioxidants-1680560-supplementary.pdf]

Table S1. Statistically significant results of seminological studies, molecular analyzes of the polymorphisms of the genes located on chromosome 5 (IL-4v.C589T (rs2243250), activity of antioxidant enzymes (superoxide dismutase SOD, catalase CAT, glutathione peroxidase GPx, and glutathione reductase GR), concentration chemical elements (Ca, Mg, Na, P, Mn, Fe, Zn, Mo, Li, V, Co, Ag, Ba, Ti, Tl, Sr, Al, Sn, Ni, B, Hg, Cd, As, Be) and the intensity of lipoperoxidation (concentration of malondialdehyde MDA) in healthy controls and men with fertility disorders.

Survey number	Group	Seminological study	IL-4 C589T	SOD [U·mL^{-1}]	CAT [nmol·min^{-1}·mL^{-1}]	GPx [nmol·min^{-1}·mL^{-1}]	GR [nmol·min^{-1}·mL^{-1}]	MDA [uM]	Ca [mg·L^{-1}]	Mg [mg·L^{-1}]	Na [mg·L^{-1}]	P [mg·L^{-1}]	Mn [mg·L^{-1}]	Fe [mg·L^{-1}]	Zn [mg·L^{-1}]	Mo [mg·L^{-1}]	Li [mg·L^{-1}]	V [mg·L^{-1}]	Co [mg·L^{-1}]	Ag [mg·L^{-1}]	Ba [mg·L^{-1}]	Ti [mg·L^{-1}]	Tl [mg·L^{-1}]	Sr [mg·L^{-1}]	Al [mg·L^{-1}]	Sn [mg·L^{-1}]	Ni [mg·L^{-1}]	B [mg·L^{-1}]	Hg [mg·L^{-1}]	Cd [mg·L^{-1}]	As [mg·L^{-1}]	Be [mg·L^{-1}]
4	Healthy	normozoospermia							57.7	40.2	1796	341	0.027	545	10.096	0.01755	0.151	0.004	0.004036	0.001	0.058	0.016	0.00261	0.028	0.953	0.062	0.008	2.438	0.02975	0.00337	0.016	0
5	Healthy	normozoospermia							50.1	38.9	1652	347	0.029	520	6.753	0.011604	0.184	0.002	0.001234	0	0.082	0.027	0.00174	0.019	1.38	0.051	0.025	2.417	0.02468	0.00388	0.013	0
6	Healthy	normozoospermia							51.7	39.4	1720	351	0.026	567	10.833	0.010723	0.183	0.003	0.00505	0	0.062	0.026	0.0014	0.011	0.748	0.044	0.019	2.24	0.02887	0.00365	0.02	0
7	Healthy	normozoospermia																														
8	Infertile	**azoospermia**							47.6	35.7	1579	354	0.015	544	9.905	0.008834	0.178	0.003	0.003444	0	0.048	0.017	0.00289	0.017	0.734	0.035	0.009	2.129	0.0027	0.02476	0.014	0.047
9	Healthy	normozoospermia							54.9	37.9	1870	340	0.023	530	9.041	0.011521	0.167	0.003	0.004494	0	0.072	0.02	0.0027	0.013	0.858	0.037	0.014	2.389	0.02297	0.00626	0.014	0
10	Healthy	normozoospermia							59.5	37.1	1941	375	0.016	548	8.836	0.008184	0.154	0.004	0.004009	0	0.092	0.023	0.00166	0.029	1.032	0.036	0.01	2.241	0.03371	0.00737	0.015	0
11	Infertile	**azoospermia**		0.393	18.1	8.321	99.5	2.454	55.7	38.8	1871	357	0.019	564	10.392	0.007768	0.195	0.004	0.004453	0	0.089	0.039	0.00175	0.014	0.937	0.034	0.015	2.044	0.02969	0.01816	0.014	0
12	Healthy	normozoospermia		0.149	130.402	5.094	20.715	28.357	43.7	35.9	1618	354	0.022	564	10.232	0.007396	0.175	0.004	0.004073	0	0.058	0.028	0.00143	0.016	0.742	0.034	0.024	1.851	0.02667	0.00475	0.015	0
13	Healthy	normozoospermia		0.382	39.1	0	18.677		47.1	36.7	1678	330	0.018	557	8.24	0.007022	0.147	0.003	0.003967	0	0.099	0.022	0.00112	0.012	0.973	0.036	0.01	1.93	0.0345	0.0053	0.015	0
14	Healthy	normozoospermia			29.061	0			55	36.5	1707	327	0.018	558	10.557	0.006101	0.217	0.005	0.00049	0	0.067	0.023	0.00153	0.027	0.949	0.055	0.018	2.074	0.01797	0.00262	0.016	0
15	Inf	**oligozoo**		0.2	40.56	1.019	19.69	16.	55.	40.	17	38	0.0	63	10.	0.0	0.1	0.0	0.0	0	0.0	0.0	0.0	0.0	1.0	0.0	0.0	1.9	0.0	0.0	0.0	0

	ertile	**spermia**		05	6		6	929	3	7	98	1	19	1	939	05229	88	06	04563		85	28	00208	19	46	59	09	36	02592	01595	17	
16	Healthy	normozoospermia		0.035	48.113	0.509	21.224	53.857	46.4	33.5	1636	340	0.019	527	6.603	0.005771	0.186	0.003	0.00656	0	0.069	0.03	0.00098	0.017	1.079	0.036	0.012	1.772	0.03077	0.00346	0.013	0
17	Healthy	normozoospermia		0.159	157.074	2.038	18.168	70.5	41.4	36.5	1700	342	0.023	590	11.652	0.008475	0.154	0.005	0.00973	0	0.064	0.028	0.00198	0.028	0.984	0.065	0.031	1.883	0.02692	0.00241	0.014	0
18	Infertile	**asthenozoospermia**		0.005	40.272	3.056	13.584	25.5	55.2	39.4	1839	334	0.018	488	8.919	0.007483	0.211	0.004	0.04373	0	0.057	0.027	0.00149	0.029	0.927	0.069	0.01	1.73	0.02448	0.00259	0.014	0
19	Healthy	normozoospermia		0.061	49.578	2.547	33.45	55.5	57.5	35.8	1737	352	0.02	567	10.702	0.004808	0.195	0.005	0.04304	0	0.068	0.042	0.00152	0.019	1.028	0.054	0.009	1.584	0.02869	0.0277	0.017	0
20	Healthy	normozoospermia		0.338	116.259	2.547	23.262	118.357	58.5	34.4	1813	334	0.025	506	6.575	0.006728	0.184	0.004	0.00647	0	0.056	0.028	0.0013	0.039	0.838	0.033	0.018	1.63	0.02232	0.0038	0.016	0.0034
21	Healthy	normozoospermia		0.169	20.195	4.075	25.809	69.786	50.9	34.9	1754	337	0.018	517	10.033	0.00426	0.216	0.005	0.00549	0	0.058	0.022	0.00131	0.022	0.88	0.053	0.012	1.607	0.03028	0.00512	0.018	0
22	Healthy	normozoospermia		0.372	25.104	0.509	18.677	94.071	55.1	37.2	1823	343	0.022	549	6.613	0.003859	0.173	0.004	0.01008	0	0.07	0.019	0.00072	0.044	1.227	0.041	0.041	1.788	0.02127	0.00392	0.014	0.017
23	Infertile	**asthenozoospermia**		0.137	30.893	5.094	12.565	40.5	48.6	39.3	1723	369	0.026	536	7.266	0.004575	0.212	0.004	0.0058	0	0.053	0.027	0.00154	0.015	0.775	0.048	0.011	1.638	0.02002	0.00266	0.014	0
24	Healthy	normozoospermia		0.43	49.872	0	19.696	7.643	53.4	36.8	1826	333	0.017	500	9.352	0.004237	0.199	0.006	0.00693	0	0.066	0.04	0.00118	0.015	0.919	0.051	0.013	1.533	0.02031	0.00548	0.017	0
25	Healthy	normozoospermia		0.488	50.971	0.509	17.659	5.5	60.3	37.1	1881	334	0.025	531	9.397	0.003207	0.137	0.004	0.04706	0	0.06	0.019	0.00163	0.023	1.017	0.07	0.011	1.634	0.01882	0.00551	0.016	0
26	Healthy	normozoospermia							61.1	36.9	1748	344	0.021	581	11.569	0.004539	0.189	0.006	0.0512	0	0.052	0.026	0.00176	0.02	1.026	0.054	0.011	1.673	0.02192	0.00655	0.016	0
27	Healthy	normozoospermia		0.058	59.178	1.019	18.677	0.214	56.2	34.3	1746	362	0.023	532	9.366	0.004147	0.194	0.004	0.00324	0	0.057	0.073	0.00218	0.017	0.817	0.062	0.008	1.731	0.02347	0.00401	0.013	0
28	Healthy	normozoospermia		0.311	41.298	3.566	11.037	0.5	49.5	34	1642	340	0.018	528	6.154	0.004252	0.165	0.003	0.00292	0	0.071	0.029	0.00162	0.012	0.885	0.049	0.01	1.531	0.01749	0.00703	0.015	0
29	He	normozo		0.0	64.6	4.584	33.45	3.0	58.	39.	18	33	0.0	55	7.1	0.0	0.1	0.0	0.0	0	0.0	0.0	0.0	0.0	0.7	0.0	0.0	1.5	0.0	0.0	0.0	0.0

	althy	ospermia		3				71	3	6	71	7	23	8	91	03327	89	04	03253		3	29	00146	21	18	66	09	28	01915	00273	11	017
30	Healthy	normozoospermia	TT	0.6	14.919	1.019	22.752	0.214	46	35.1	1745	348	0.018	513	6.02	0.002543	0.158	0.003	0.000324	0	0.049	0.02	0.000131	0.015	0.63	0.033	0.01	1.429	0.001877	0.000487	0.013	0
31	Infertile	**Combined**	TT	0.239	23.859	0.509	19.187	1.214	57.4	40.6	1585	361	0.022	580	8.403	0.003329	0.208	0.004	0.003943	0	0.061	0.021	0.000101	0.013	0.708	0.067	0.008	1.544	0.002015	0.001669	0.014	0
32	Healthy	normozoospermia		0.377	28.622	0.509	21.734	32.643	47.3	38.4	1783	345	0.022	559	8.341	0.002177	0.209	0.004	0.003867	0.002	0.042	0.019	0.000131	0.01	0.642	0.063	0.018	1.481	0.001731	0.000126	0.014	0
34	Infertile	**OAT**		0.593	27.742	0.509	19.696	3.357	49	36.2	1937	314	0.028	528	8.449	0.004221	0.225	0.004	0.002628	0	0.372	0.028	0.000129	0.032	2.009	0.079	0.014	1.616	0.002023	0.000434	0.014	0
35	Healthy	normozoospermia	TT	0.615	66.359	0.509	20.715		44.5	36.1	1731	341	0.014	522	12.326	0.006564	0.208	0.004	0.000579	0	0.62	0.023	0.000059	0.042	0.618	0.062	0.01	1.474	0.002328	0.000234	0.017	0
36	Infertile	**polyzoospermia**		0.66	63.794	0.509	21.224		52.4	33	1676	309	0.023	535	9.124	0.003847	0.156	0.003	0.000437	0	0.378	0.027	0.000084	0.024	0.665	0.241	0.011	1.566	0.002402	0.000207	0.013	0
37	Healthy	normozoospermia		0.073	66.212	3.056	21.055		55.3	32.9	1745	337	0.015	520	9.015	0.007054	0.183	0.004	0.000451	0	0.341	0.023	0.000172	0.021	0.777	0.064	0.009	1.421	0.001964	0.000521	0.016	0
38	Infertile	**asthenozoospermia**	TT	0.565	24.225	0	19.696	18.357	48.6	34.4	1893	333	0.02	480	10.225	0.008894	0.21	0.002	0.000355	0	0.409	0.027	0.00019	0.031	0.773	0.074	0.035	1.627	0.004444	0.000275	0.009	0.0017
39	Healthy	normozoospermia		0.205	37.122	6.113	29.374	3.786	54.9	35.5	1715	339	0.016	539	9.762	0.008875	0.196	0.002	0.000353	0	0.38	0.043	0.000116	0.028	0.901	0.065	0.018	1.379	0.001891	0.000296	0.009	0
40	Healthy	normozoospermia		0.088	41.298	4.075	18.168	2.643	49.5	35.7	1794	336	0.022	561	8.85	0.005515	0.193	0.004	0.000385	0	0.405	0.032	0.000087	0.028	0.618	0.047	0.01	1.445	0.001766	0.000378	0.011	0
41	Healthy	normozoospermia	CC	0.408	52.216	1.019	23.262	15.5	51.9	33.8	1746	340	0.025	540	8.921	0.005879	0.172	0.003	0.000229	0	0.351	0.035	0.000088	0.027	0.792	0.044	0.008	1.394	0.001806	0.000234	0.012	0.0017
42	Healthy	normozoospermia		0.43	23.932	0.509	12.904	19.571	40.6	33	1666	323	0.017	492	8.729	0.005004	0.2	0.002	0.001746	0	0.35	0.014	0.000145	0.023	0.836	0.037	0.036	1.417	0.002475	0.000294	0.017	0
43	Healthy	normozoospermia	CC	0.297	39.686	0	11.886		43.8	41.3	1667	350	0.024	541	8.319	0.004278	0.215	0.003	0.000399	0	0.279	0.019	0.000083	0.021	0.669	0.036	0.011	1.349	0.002662	0.000298	0.012	0
44	Inf	**Combin**	C	0.6	176.9	1.019	15.62	209	47	30.	17	29	0.0	47	10.	0.0	0.2	0.0	0.0	0	0.3	0.0	0.0	0.0	0.6	0.0	0.0	1.3	0.0	0.0	0.0	0

	ertile	**ed**	C	68	32		1	.786		4	88	0	18	9	412	05975	01	03	02666		1	2	00164	22	46	37	12	44	02081	00578	13	
45	Healthy	normozoospermia	TT	0.002	82.186	9.678	31.921	3.786	54.7	32.5	1728	318	0.016	509	8.305	0.005072	0.221	0.004	0.000375	0	0.304	0.022	0.00046	0.018	0.63	0.038	0.007	1.202	0.001792	0.000155	0.012	0
46	Healthy	normozoospermia	TT	1.242	27.156	1.019	14.093	12.357	48	31.7	1627	309	0.024	518	8.462	0.003009	0.203	0.004	0.000412	0	0.332	0.018	0.000108	0.045	0.714	0.042	0.152	1.194	0.003344	0.000265	0.013	0.018
47	Infertile	**oligozoospermia**		0.136	40.859	1.019	14.942	0.929	58.6	36	1850	352	0.015	487	7.977	0.004662	0.196	0.004	0.00047	0	0.323	0.032	0.000122	0.027	0.676	0.039	0.022	1.267	0.001677	0.00033	0.012	0
48	Healthy	normozoospermia	TT	0.103	49.432	0.509	25.299	124.786	51.4	37.1	1671	380	0.025	543	8.898	0.004172	0.165	0.003	0.001875	0.001	0.295	0.023	0.000195	0.022	0.585	0.039	0.014	1.279	0.001663	0.000349	0.012	0.018
49	Infertile	**OAT**	TT	0.425	37.195	2.038	17.149	6.214	46	36.1	1767	342	0.019	502	9.208	0.004391	0.182	0.004	0.000455	0	0.35	0.025	0.000125	0.05	0.668	0.055	0.007	1.187	0.003571	0.000327	0.013	0
50	Healthy	normozoospermia	TT	0.552	22.759	1.528	14.093	1.643	58.2	38.3	1762	353	0.024	548	9.681	0.003191	0.182	0.005	0.001165	0	0.333	0.02	0.000208	0.03	0.714	0.038	0.037	1.275	0.00181	0.000435	0.015	0
51	Healthy	normozoospermia	TT	0.579	55.147	0.509	15.621	94.786	51.5	39.9	1819	363	0.02	555	9.235	0.003386	0.218	0.002	0.00249	0	0.306	0.025	0.000063	0.025	0.806	0.038	0.024	1.281	0.001754	0.000229	0.013	0
52	Healthy	normozoospermia	TT	0.545	36.609	0	16.64	6.643	43.1	35.3	1615	333	0.014	570	9.207	0.00353	0.188	0.004	0.00222	0	0.31	0.088	0.000059	0.014	0.551	0.041	0.01	1.29	0.001697	0.000411	0.014	0
53	Healthy	normozoospermia		0.436	32.578	2.038	22.752	4.071	62.6	36	1703	369	0.015	548	7.763	0.003219	0.234	0.005	0.000267	0	0.358	0.022	0.000104	0.024	0.751	0.057	0.019	1.237	0.001996	0.000303	0.016	0
54	Healthy	normozoospermia	TT	0.284	34.777	1.528	31.921	30.5	53.7	32.6	1979	324	0.017	497	8.5	0.005617	0.195	0.006	0.000542	0	0.346	0.021	0.00003	0.054	0.602	0.057	0.069	1.239	0.001504	0.000462	0.016	0
55	Healthy	normozoospermia	TT	0.348	52.583	1.528	16.13	1.214	53.6	41	1939	378	0.018	615	8.326	0.004068	0.196	0.005	0.0023	0	0.37	0.018	0.000033	0.022	0.662	0.063	0.011	1.181	0.001422	0.00023	0.017	0
56	Infertile	**Combined**		0.069	51.777	3.566	24.281	1.214	58.2	37.4	1635	358	0.019	543	11.975	0.004059	0.17	0.005	0.004277	0	0.368	0.032	0.000073	0.039	0.642	0.057	0.008	1.164	0.003159	0.00036	0.017	0
58	Infertile	**asthenozoospermia**		0.836	59.691	2.038	17.149	5.929	54	33.3	1860	317	0.118	519	11.209	0.038231	0.168	0.007	0.013302	0	0.403	0.027	0.000106	0.021	0.702	0.055	0.355	1.2	0.001598	0.000394	0.016	0
59	He	normozo	TT	0.5	39.68	0.509	18.16	5.9	42.	34.	18	32	0.0	56	8.9	0.0	0.1	0.0	0.0	0	0.2	0.0	0.0	0.0	0.6	0.0	0.0	1.2	0.0	0.0	0.0	0

	althy	ospermia		59	6		8	29	4	6	24	8	2	7	5	06735	98	03	01905		96	26	00072	16	6	62	07	27	0229	00253	1	
60	Healthy	normozoospermia	CC	0.284	113.988	0	2.377		56	36.4	1860	330	0.018	529	10.471	0.007312	0.175	0.004	0.000322	0	0.417	0.015	0.000133	0.024	0.732	0.067	0.009	1.296	0.002098	0.000299	0.014	0
61	Infertile	**asthenozoospermia**	TT	0.393	36.462	2.038	23.771	9.786	46	37.1	1733	360	0.018	545	8.699	0.004512	0.228	0.003	0.002401	0	0.299	0.031	0.000101	0.028	0.586	0.059	0.008	1.288	0.001869	0.000329	0.01	0
62	Healthy	normozoospermia	TT	0.093	86.876	1.019	23.771	0.5	48.8	39.5	1559	388	0.019	614	9.724	0.004833	0.191	0.004	0.00043	0	0.292	0.028	0.000164	0.031	0.615	0.06	0.009	1.199	0.002511	0.001062	0.012	0
63	Healthy	normozoospermia		0.752	40.272	0.509	20.206	5.5	51.4	39.5	1908	369	0.018	540	8.886	0.00468	0.223	0.005	0.001666	0	0.377	0.023	0.000182	0.033	0.634	0.061	0.007	1.236	0.002092	0.000707	0.013	0.019
64	Infertile	**OAT**	TT	0.5	142.492	1.528	23.771	3.357	58.4	41.7	1770	375	0.016	569	8.566	0.003091	0.21	0.003	0.002023	0	0.305	0.04	0.000043	0.016	0.834	0.047	0.009	1.278	0.001824	0.000188	0.01	0
65	Infertile	**OAT**	TT	0.66	30.014	0	19.187	3.786	48.4	32.8	1766	310	0.02	496	9.68	0.004556	0.195	0.004	0.000423	0	0.354	0.019	0.000119	0.106	0.821	0.047	0.009	1.203	0.001837	0.000539	0.015	0
66	Infertile	**asthenozoospermia**	TT	1.112	144.691	1.019	18.677	1.643	50	30.8	1762	344	0.016	526	8.728	0.004154	0.17	0.004	0.000532	0	0.405	0.038	0.00009	0.024	0.721	0.044	0.01	1.154	0.004962	0.000436	0.013	0
67	Infertile	**polyzoospermia**	TT	0.545	76.764	3.056	21.734	0.214	49.2	33.4	1670	350	0.018	561	9.073	0.004307	0.226	0.004	0.002967	0	0.333	0.035	0.000089	0.035	0.749	0.045	0.009	1.195	0.002559	0.000497	0.015	0
68	Healthy	normozoospermia	TT	0.377	52.143	1.528	19.187	6.643	54.7	38.3	1964	370	0.029	544	11.194	0.011158	0.185	0.005	0.003768	0	0.418	0.029	0.00015	0.131	0.808	0.06	0.012	1.24	0.002377	0.000454	0.015	0
69	Infertile	**azoospermia**		0.01	74.859	21.903	3.396	13.357																								
70	Healthy	normozoospermia		0.055	29.794	2.038	20.206	33.357	55.3	35.9	1859	357	0.019	521	7.816	0.002829	0.172	0.002	0.002176	0.001	0.372	0.088	0.00003	0.033	0.691	0.057	0.01	1.296	0.001394	0.000214	0.013	0
71	Healthy	normozoospermia	TT	0.579	48.04	0.509	17.149	7.357	56.9	37.4	1812	351	0.013	529	8.666	0.004226	0.192	0.005	0.000413	0	0.307	0.021	0.000187	0.03	0.717	0.056	0.008	1.193	0.001741	0.000374	0.013	0
72	Infertile	**OAT**	TT	0.311	38.807	0	27.846	6.929	41.5	37.4	1810	363	0.018	602	9.609	0.00512	0.197	0.005	0.000673	0	0.275	0.024	0.0001	0.019	0.505	0.056	0.01	1.117	0.001427	0.00042	0.013	0
73	He	normozo	TT	0.8	45.91	1.019	22.75	0.5	55.	39.	17	35	0.0	56	9.8	0.0	0.2	0.0	0.0	0	0.3	0.0	0.0	0.0	0.4	0.0	0.0	1.2	0.0	0.0	0.0	0.0

	althy	ospermia		76	5		2		9	2	36	5	16	9	2	045	28	04	00443		5	95	00102	39	64	64	09	19	03935	01054	12	034
74	Healthy	normozoospermia		0.251	13.527	2.038	25.299	7.643	60.8	34.2	1732	325	0.015	531	9.54	0.004089	0.194	0.005	0.002343	0	0.335	0.023	0.00115	0.031	0.536	0.06	0.011	1.136	0.02268	0.00462	0.011	0
75	Healthy	normozoospermia	CC	0.324	45.988	1.019	10.867	5.5	53.9	28.8	1724	311	0.012	485	8.766	0.003165	0.157	0.003	0.00557	0	0.291	0.029	0.00068	0.016	0.486	0.051	0.012	1.153	0.01464	0.00219	0.011	0
76	Healthy	normozoospermia	TT	0.227	27.229	1.019	18.677	5.214	54.2	41.2	1785	384	0.016	621	10.267	0.004115	0.161	0.004	0.00161	0	0.34	0.013	0.00206	0.026	0.66	0.061	0.009	1.175	0.01621	0.00485	0.011	0
77	Healthy	normozoospermia		0.017	69.143	3.566	19.696	6.643	54.4	38.7	1940	328	0.013	490	8.83	0.005846	0.202	0.004	0.00646	0	0.365	0.03	0.00104	0.027	0.344	0.075	0.008	1.065	0.01805	0.00299	0.014	0
78	Healthy	normozoospermia		0.436	78.816	3.056	20.206	11.214	60.2	32.7	2287	332	0.027	468	9.852	0.003152	0.172	0.004	0.00655	0	0.401	0.016	0.00352	0.038	0.862	0.061	0.012	1.149	0.02077	0.00277	0.013	0
79	Healthy	normozoospermia	TT	0.097	21.367	1.019	27.337	1.643	54.2	33.6	1754	342	0.015	514	7.813	0.003145	0.19	0.004	0.00546	0	0.316	0.028	0.00162	0.016	0.909	0.059	0.01	1.148	0.02196	0.00252	0.014	0
80	Healthy	normozoospermia	TT	0.343	10.596	5.094	21.224	242.643	44.8	33.3	1736	323	0.016	525	8.877	0.003227	0.226	0.005	0.0032	0	0.297	0.022	0.00073	0.024	0.755	0.057	0.011	1.133	0.01159	0.00333	0.013	0
81	Healthy	normozoospermia	TT	0.513	7.078	4.584	21.734	4.5	54.3	31.9	1849	307	0.021	467	7.689	0.007454	0.216	0.002	0.00608	0	0.32	0.035	0.00044	0.037	0.677	0.058	0.012	1.245	0.02196	0.00212	0.009	0
82	Healthy	normozoospermia		0.377	22.54	4.584	21.224	2.357	47.1	41	1887	392	0.037	533	8.669	0.269966	0.208	0.016	0.007414	0	0.274	0.04	0.00146	0.046	0.552	0.067	0.239	1.179	0.02012	0.00295	0.012	0
82	Healthy	normozoospermia							54.3	33.2	1908	340	0.017	503	9.313	0.00557	0.178	0.004	0.00329	0	0.328	0.022	0.00224	0.021	0.819	0.057	0.009	1.143	0.01462	0.00304	0.011	0
83	Infertile	**asthenozoospermia**		0.887	4.074	4.584	19.187	0.929	58.2	32.7	2006	290	0.016	415	7.531	0.005139	0.203	0.004	0.00506	0	0.3	0.025	0.00058	0.02	0.492	0.06	0.008	1.164	0.02084	0.00229	0.013	0.0017
84	Infertile	**Combined**		0.408	3.561	2.038	19.187	76.214	46.9	36	1752	341	0.022	540	9.252	0.003377	0.225	0.004	0.01574	0	0.279	0.023	0.00089	0.027	0.76	0.08	0.012	1.139	0.02723	0.00456	0.017	0.0018
85	Healthy	normozoospermia		0.615	5.173	5.094	20.206	3.071	53.9	38.1	1794	351	0.018	524	9.378	0.003685	0.253	0.003	0.00445	0.001	0.304	0.019	0.00132	0.02	0.631	0.066	0.015	1.01	0.02012	0.00336	0.014	0.0018
86	Inf	**cryptozo**							51.	37.	17	34	0.0	52	9.9	0.0	0.1	0.0	0.0	0	0.3	0.0	0.0	0.0	0.8	0.0	0.0	1.1	0.0	0.0	0.0	0.0

	ertile	**ospermia**							7	9	91	4	12	1	19	02766	76	04	00528		45	2	00119	33	35	62	1	11	02114	00347	13	018
87	Healthy	normozoospermia		0.653	13.453	6.622	24.281	7.357	58.5	34.7	1929	377	0.016	537	10.025	0.007324	0.216	0.005	0.000673	0	0.352	0.024	0.00016	0.062	0.782	0.063	0.036	1.188	0.002714	0.000567	0.014	0
88	Healthy	normozoospermia		0.559	2.462	9.678	51.618	126.929	45.1	35.2	1650	323	0.018	529	8.924	0.004611	0.185	0.004	0.000486	0	0.318	0.023	0.000059	0.019	0.707	0.061	0.008	1.054	0.00222	0.000301	0.016	0
89	Infertile	**azoospermia**	TT	1.035	26.35	3.566	17.149	138.357	59.2	33.4	2105	325	0.021	341	7.135	0.003746	0.21	0.004	0.000227	0	0.292	0.015	0.000146	0.024	0.634	0.058	0.008	1.221	0.001696	0.001541	0.014	0
90	Infertile	**OAT**	TT	0.176	37.854	1.528	17.659	6.643	62.4	34	1998	322	0.017	457	8.164	0.003834	0.179	0.004	0.000389	0	0.321	0.017	0.000117	0.023	0.729	0.056	0.009	1.172	0.002386	0.000214	0.013	0
91	Infertile	**OAT**	TT	0.692	66.285	2.547	19.187	2.357	59.4	35.3	1765	316	0.024	529	9.13	0.00232	0.165	0.005	0.000426	0	0.305	0.017	0.000044	0.019	0.825	0.077	0.011	1.079	0.002833	0.000389	0.012	0
92	Infertile	**necrozoospermia**	TT	0.464	41.665	3.056	22.752	16.929	48.7	37.6	1880	356	0.018	500	8.453	0.003378	0.198	0.004	0.000263	0	0.325	0.023	0.00015	0.023	0.671	0.064	0.008	1.061	0.001677	0.000455	0.013	0
93	Healthy	normozoospermia	CC	0.684	26.57	4.075	23.262	38.357	56	32.2	2061	299	0.021	443	7.74	0.002877	0.208	0.004	0.000762	0	0.347	0.025	0.000096	0.033	0.785	0.08	0.008	1.109	0.001484	0.000251	0.014	0
94	Healthy	normozoospermia	CC	0.734	45.988	6.113	18.677	1.806	59	33.1	2239	330	0.016	507	9.327	0.003145	0.244	0.004	0.000433	0	0.33	0.02	0.000121	0.03	0.773	0.066	0.012	1.107	0.001776	0.000263	0.015	0
95	Infertile	**azoospermia**	TT	0.615	70.207	6.622	19.187	3.935	63.1	31.7	2042	331	0.021	420	7.864	0.005118	0.156	0.004	0.001874	0	0.345	0.025	0.000149	0.074	0.917	0.065	0.012	1.093	0.001969	0.000667	0.014	0
96	Healthy	normozoospermia	TT	0.124	28.987	0.509	19.696		45.6	41.7	1730	430	0.019	702	10.76	0.004149	0.204	0.005	0.000443	0	0.338	0.022	0.000148	0.032	1.117	0.075	0.009	1.115	0.002076	0.00097	0.016	0.002
97	Healthy	normozoospermia	TT	0.919	23.515	3.566	19.187	2.731	54.5	36.7	1922	363	0.03	559	8.815	0.002757	0.166	0.005	0.00043	0.001	0.32	0.022	0.000075	0.03	0.828	0.06	0.01	1.051	0.001922	0.000087	0.016	0
98	Infertile	**oligozoospermia**	TT			2.038		46.714	53.2	38.7	1892	359	0.018	538	8.149	0.003157	0.193	0.004	0.002068	0	0.307	0.02	0.0001	0.037	0.785	0.067	0.025	1.179	0.001719	0.001605	0.013	0
99	Infertile	**OAT**	CC	0.565	23.806	2.547	17.659	42.429	43.1	34.6	1710	334	0.014	558	9.652	0.003968	0.164	0.004	0.001982	0	0.283	0.019	0.000099	0.02	0.289	0.076	0.01	1.063	0.001702	0.000312	0.012	0
100	Inf	**azoosper**		0.2	37.89	1.019	32.43	3.7	50.	32.	19	34	0.0	48	8.9	0.0	0.2	0.0	0.0	0	0.3	0.0	0.0	0.0	0.5	0.0	0.0	1.0	0.0	0.0	0.0	0

	ertile	**mia**		22	5		1	5	5	8	02	0	2	8	91	01783	06	05	00517		01	24	00145	2	19	62	25	61	02115	00191	13	
101	Infertile	**oligozoospermia**	CC	0.402	30.85	6.113	17.149	3.009	56.2	36.5	2039	317	0.013	442	8.276	0.008806	0.183	0.003	0.002474	0	0.332	0.022	0.000102	0.027	0.537	0.074	0.006	0.996	0.002165	0.00023	0.012	0
102	Infertile	**Combined**	TT	0.323	36.789	8.15	24.281	3.38	50.7	38.6	1896	364	0.017	570	11.28	0.004979	0.174	0.003	0.003854	0	0.321	0.021	0.000132	0.012	0.696	0.078	0.013	1.204	0.002542	0.00294	0.016	0
104	Infertile	**Combined**		0.258	46.744	3.566	25.299	1.806	44.3	36.4	1479	356	0.019	660	10.074	0.003887	0.193	0.005	0.002356	0	0.298	0.018	0.000129	0.024	0.594	0.064	0.008	0.958	0.001321	0.00685	0.012	0.0017
105	Healthy	normozoospermia		0.686	40.049	8.66	0.17	10.417	60.3	32	2081	340	0.015	479	8.73	0.004546	0.226	0.003	0.000329	0	0.44	0.022	0.000183	0.059	0.581	0.077	0.007	1.075	0.002249	0.00462	0.013	0
107	Infertile	**Combined**		0.402	39.641	22.242	18.508	6.157	55.2	41.9	1842	387	0.018	612	8.704	0.005014	0.213	0.004	0.000373	0	0.363	0.024	0.000169	0.025	0.711	0.076	0.02	1.117	0.001596	0.01945	0.013	0
108	Infertile	**Combined**		0.337	44.008	4.755	31.921	6.157	46.6	37.1	1806	363	0.015	570	8.663	0.005315	0.175	0.005	0.000262	0	0.295	0.019	0.000136	0.022	0.569	0.081	0.007	1.049	0.002168	0.06057	0.015	0
109	Infertile	**asthenozoospermia**		0.03	41.504	8.321	19.187	9.12	50.5	38.2	1832	371	0.023	543	8.099	0.003797	0.241	0.004	0.000708	0	0.32	0.021	0.000123	0.018	0.676	0.064	0.01	1.118	0.002814	0.00266	0.012	0
110	Infertile	**OAT**		0.712	20.895	11.377	20.206	1.991	52.6	34.8	1981	339	0.022	556	8.393	0.005404	0.137	0.002	0.002699	0.001	0.317	0.022	0.000135	0.039	0.701	0.062	0.014	1.205	0.001763	0.00282	0.012	0
111	Healthy	normozoospermia		0.622	15.306	11.886	13.074	1.343	55.9	40.1	1882	349	0.019	596	7.014	0.003921	0.19	0.003	0.002305	0	0.317	0.027	0.000139	0.019	0.671	0.055	0.007	1.02	0.002918	0.00531	0.011	0.0018
112	Infertile	**Combined**	TT	0.982	20.662	11.886	15.621	2.269	52.6	38.2	1970	333	0.016	553	8.516	0.004366	0.228	0.004	0.003032	0	0.318	0.016	0.000105	0.014	0.439	0.056	0.009	1.037	0.002259	0.00306	0.01	0
113	Healthy	normozoospermia		0.706	23.165	11.377	10.357	4.491	51.1	35.6	1862	333	0.016	511	7.437	0.005069	0.236	0.003	0.00193	0	0.283	0.021	0.000089	0.035	0.746	0.059	0.008	1.11	0.001623	0.00389	0.011	0.0018
114	Healthy	normozoospermia	TT	0.739	34.169	10.868	14.433	0.694	45.4	38.9	1655	364	0.026	605	9.052	0.003946	0.209	0.003	0.000331	0	0.302	0.019	0.000228	0.022	0.554	0.057	0.007	1.115	0.001756	0.00308	0.011	0
115	Healthy	normozoospermia	CC	0.728	92.796	11.377	19.017	3.287	47.2	35.8	1869	323	0.022	574	8.608	0.004291	0.205	0.003	0.002493	0	0.315	0.039	0.000136	0.021	0.516	0.066	0.009	1.055	0.001471	0.00375	0.009	0
116	He	normozo	TT	0.6	12.68	11.88	16.47	4.8	51.	34.	19	31	0.0	49	9.4	0.0	0.1	0.0	0.0	0	0.3	0.0	0.0	0.0	0.5	0.0	0.0	1.0	0.0	0.0	0.0	0

	althy	ospermia		61	6	6		61	2	1	92	3	21	2	04	03286	89	05	00546		42	3	00133	21	25	57	09	9	01426	00275	09	
117	Infertile	**asthenozoospermia**	TT	0.613	26.309	10.868	1.868	4.954	51.4	35.1	1983	337	0.021	541	9.307	0.003973	0.208	0.003	0.000442	0	0.356	0.118	0.00073	0.02	0.986	0.062	0.008	1.007	0.001919	0.000358	0.01	0
119	Infertile	**oligozoospermia**	CC	0.276	23.457	10.358	12.904	0.694	53.8	34.7	1963	339	0.014	522	7.189	0.003117	0.219	0.003	0.0022	0	0.362	0.024	0.0012	0.014	0.589	0.054	0.008	1.056	0.001407	0.000282	0.01	0.0018
120	Infertile	**oligozoospermia**	CC	0.573	13.85	12.396	13.923	1.806	53.6	36.1	1783	332	0.016	563	9.767	0.003213	0.194	0.005	0.002628	0.001	0.344	0.036	0.000208	0.02	1.035	0.053	0.008	0.969	0.001697	0.000748	0.011	0
121	Infertile	**Combined**	CC	0.436	30.093	10.868	24.111	1.62	58.3	36.5	1797	358	0.016	551	8.328	0.003524	0.191	0.004	0.00176	0	0.393	0.026	0.000076	0.027	0.798	0.046	0.007	1.106	0.001689	0.002919	0.012	0
122	Infertile	**Combined**	TT	0.331	35.391	4.755	17.998	5.88	58.2	34.1	1735	308	0.02	500	8.636	0.006528	0.189	0.003	0.000508	0	0.359	0.029	0.000233	0.028	0.813	0.048	0.032	1.057	0.002709	0.000273	0.01	0.0033
123	Infertile	**Combined**	TT	1.02	145.95	11.377	3.735	2.917																								
124	Infertile	**asthenozoospermia**		0.784	44.939	9.849	6.282	1.62																								
125	Infertile	**azoospermia**		0.595	19.963	5.774	8.32	3.75																								
126	Infertile	**Combined**		0.547	24.912	11.377	7.301	3.009																								
127	Healthy	normozoospermia		0.877	5.467	12.396	2.717	1.157	60.5	33.7	1917	313	0.018	468	8.883	0.006467	0.221	0.004	0.000355	0	0.448	0.04	0.000225	0.031	0.852	0.046	0.007	0.955	0.00305	0.000322	0.011	0
129	Healthy	normozoospermia		0.87	16.179	11.377	10.357	6.25																								
130	Infertile	**Combined**	CC	0.681	17.46	11.886	14.433	4.769	54.8	37.7	1892	327	0.013	526	12.264	0.005471	0.232	0.004	0.004338	0	0.345	0.026	0.000223	0.023	0.865	0.051	0.007	0.994	0.003158	0.000645	0.013	0
131	Healthy	normozoospermia	TT	0.396	25.902	6.793	1.698	2.269	58.1	34.3	1945	318	0.02	490	8.398	0.005676	0.121	0.004	0.001852	0	0.489	0.018	0.000091	0.028	1.275	0.047	0.042	1.004	0.001544	0.000372	0.011	0.0017
132	He	normozo	TT	0.9	96.11	9.34	9.339	15.	52.	36.	18	31	0.0	52	9.1	0.0	0.2	0.0	0.0	0	0.3	0.0	0.0	0.0	1.3	0.0	0.0	1.0	0.0	0.0	0.0	0

	althy	ospermia		38	4			093	5	7	20	0	18	7	23	03341		04	00457		74	34	0015	22	15	46	07	71	01469	00323	1	
133	Infertile	**Combined**		0.717	23.165	8.83	10.357	1.528	50.6	37.9	1653	356	0.022	555	8.25	0.004638	0.189	0.004	0.000591	0	0.392	0.021	0.000167	0.034	0.812	0.044	0.007	0.991	0.003161	0.000417	0.012	0
135	Infertile	**OAT**	CC	0.904	16.528	10.358	2.717	0.602	59.7	33	1911	334	0.019	551	7.846	0.005203	0.189	0.005	0.000626	0.001	0.333	0.026	0.000134	0.047	0.97	0.046	0.011	1.018	0.002017	0.000683	0.012	0
136	Infertile	**OAT**		0.706	17.751	11.886	9.848	1.62	50.2	35.3	1652	389	0.019	567	9.647	0.002723	0.214	0.003	0.0034	0	0.297	0.018	0.000147	0.045	2.657	0.048	0.009	1.03	0.001574	0.000421	0.012	0
137	Infertile	**asthenozoospermia**	CC	0.306	36.847	10.868	7.811		51	39.8	1604	379	0.016	609	6.12	0.004173	0.187	0.003	0.00047	0	0.306	0.021	0.000091	0.017	0.99	0.03	0.026	0.928	0.001599	0.002062	0.013	0
138	Infertile	**OAT**	TT	0.613	26.018	10.868	4.754	0.787	51.4	33.1	1685	342	0.016	576	8.386	0.003429	0.219	0.004	0.00166	0	0.38	0.024	0.000208	0.022	1.256	0.078	0.006	1.006	0.001936	0.000345	0.013	0.0017
139	Infertile	**Combined**	TT	0.712	32.073	12.396	5.264	1.435	56.6	36.7	1840	340	0.025	556	7.471	0.003345	0.205	0.004	0.001145	0	0.351	0.018	0.000075	0.025	1.039	0.032	0.031	1.013	0.001501	0.000302	0.012	0.018
140	Healthy	normozoospermia	TT	0.665	20.604	11.377	14.433	15.231	56.6	37.3	1890	337	0.022	514	8.46	0.004277	0.179	0.005	0.002256	0	0.366	0.029	0.000145	0.022	1.09	0.049	0.019	1.001	0.001458	0.000274	0.011	0
144	Healthy	normozoospermia	TT	0.701	63.511	10.868	14.433	1.157	42.3	36.4	1853	332	0.021	523	9.062	0.0031	0.18	0.003	0.002626	0	0.35	0.032	0.000256	0.022	1.299	0.028	0.008	0.921	0.002326	0.000413	0.015	0
145	Infertile	**azoospermia**	TT	0.696	19.148	0.848	4.245	2.083	71.9	34.7	2203	339	0.016	428	8.634	0.024833	0.139	0.004	0.001962	0	0.38	0.016	0.000121	0.035	1.057	0.026	0.022	1.006	0.002002	0.000291	0.016	0
146	Infertile	**asthenozoospermia**		0.641	89.535		13.414	1.713	58.4	43.9	1777	405	0.02	545	8.318	0.00379	0.182	0.002	0.002403	0	0.394	0.021	0.000153	0.022	1.01	0.027	0.007	0.866	0.001686	0.000401	0.014	0
147	Infertile	**polyzoospermia**	CC	0.877	64.326	8.321	3.226	2.639	58.2	35.2	2079	343	0.018	548	8.556	0.003125	0.204	0.004	0.002212	0	0.365	0.016	0.000156	0.023	0.999	0.025	0.008	0.919	0.001711	0.0002	0.016	0
148	Infertile	**azoospermia**	TT	1.788	64.501	9.849	14.942	1.713	64.7	36.4	2151	354	0.025	427	7.333	0.006887	0.186	0.001	0.001507	0	0.346	0.023	0.000135	0.031	1.08	0.037	0.054	0.977	0.003136	0.000417	0.018	0
150	Healthy	normozoospermia	TT	0.503	86.391	11.886	11.886	8.426	56.6	39.1	1648	379	0.019	616	9.461	0.003603	0.221	0.003	0.000565	0	0.337	0.027	0.000074	0.034	1.071	0.026	0.009	0.96	0.002088	0.000593	0.013	0
151	Inf	**OAT**	TT	0.5	9.193	6.283	16.97	7.0	47	41.	16	41	0.0	67	9.1	0.0	0.1	0.0	0.0	0	0.3	0.0	0.0	0.0	0.9	0.0	0.0	0.9	0.0	0.0	0.0	0

	ertile			95			9	83		1	47	4	28	5	07	04159	57	02	00559		46	31	0006	13	98	29	27	22	02147	00414	13	
152	Healthy	normozoospermia	TT	0.952	70.556	10.358	12.904	1.25	54.7	36.3	1805	374	0.024	545	10.378	0.004237	0.19	0.004	0.001251	0	0.376	0.024	0.00061	0.032	1.146	0.028	0.035	1.033	0.002547	0.00491	0.014	0
153	Healthy	normozoospermia	TT	0.447	53.498	6.283	9.339	2.824	65	35.7	1879	328	0.014	475	9.326	0.004754	0.16	0.003	0.000328	0	0.343	0.028	0.00164	0.027	0.986	0.024	0.009	0.891	0.001852	0.00369	0.012	0
154	Infertile	**oligozoospermia**	TT	0.419	29.453	6.283	14.942	2.083	57.1	35	1804	308	0.029	526	8.181	0.003056	0.174	0.003	0.002674	0	0.318	0.033	0.00192	0.024	1.153	0.026	0.016	0.902	0.002734	0.00234	0.014	0
155	Healthy	normozoospermia	CC	0.82	10.357	11.886	11.886	2.176	51	34.6	1827	324	0.017	541	7.971	0.004868	0.181	0.003	0.00036	0	0.346	0.025	0.00106	0.023	1.014	0.028	0.017	0.895	0.00125	0.00879	0.014	0
156	Infertile	**Combined**	TT	0.826	19.614	8.83	10.867	2.824	64.4	41.7	1876	355	0.022	550	9.23	0.003468	0.232	0.004	0.000486	0	0.391	0.028	0.00162	0.024	1.265	0.055	0.013	1.088	0.00401	0.00361	0.014	0
158	Infertile	**cryptozoospermia**	TT	0.217	92.446	10.868	4.754	3.843	61.6	34.6	1856	365	0.02	528	8.133	0.003427	0.176	0.003	0.000415	0	0.335	0.02	0.00118	0.024	1.074	0.024	0.007	0.871	0.001808	0.00512	0.013	0
159	Infertile	**Combined**		0.838	116.898	11.886	11.376	2.639																								
160	Infertile	**Combined**		0.219	98.617	9.849	6.282	6.898	49.7	31	1760	334	0.021	539	8.245	0.003496	0.182	0.003	0.001778	0	0.353	0.031	0.00144	0.02	1.399	0.06	0.01	0.983	0.001683	0.0037	0.013	0
162	Infertile	**OAT**	TT						51.1	36.4	2049	369	0.022	511	7.808	0.002613	0.22	0.004	0.002088	0	0.39	0.027	0.00119	0.023	1.181	0.054	0.012	1.016	0.001652	0.00527	0.012	0
163	Healthy	normozoospermia	TT						58.7	37.6	2037	352	0.024	567	10.047	0.003464	0.211	0.003	0.002714	0	0.388	0.043	0.0014	0.031	1.284	0.586	0.014	1.109	0.001992	0.00292	0.012	0
164	Infertile	**asthenozoospermia**							75.1	36.7	2068	304	0.028	505	8.875	0.003214	0.205	0.005	0.000425	0	0.419	0.025	0.00104	0.038	1.441	0.056	0.015	0.948	0.0022	0.00217	0.014	0
165	Healthy	normozoospermia	TT	0.632	49.713	0.68	10.867	2.917	53	33.9	1978	357	0.016	554	8.942	0.004417	0.208	0.004	0.002137	0	0.352	0.082	0.00152	0.025	1.261	0.052	0.009	1.026	0.002444	0.00399	0.014	0
166	Infertile	**asthenozoospermia**	TT	0.838	31.84	4.755	2.717	1.806	57	39.2	1881	337	0.025	597	9.594	0.003395	0.198	0.004	0.002407	0	0.431	0.016	0.0003	0.073	1.603	0.056	0.012	1.061	0.001763	0.00393	0.011	0.0018
167	Inf	**Combin**	C	0.4	48.43	9.849	12.39	2.0	52.	40	17	33	0.0	53	8.3	0.0	0.2	0.0	0.0	0	0.3	0.0	0.0	0.0	1.3	0.0	0.0	0.8	0.0	0.0	0.0	0

	ertile	**ed**	C	99	3		5	83	8		79	9	18	8	79	03569	21	04	02162		57	21	0015	29	39	52	18	9	01424	00481	15	
168	Infertile	**azoospermia**		0.361	27.939	10.358	12.395	2.361	60.6	40.7	1825	365	0.022	555	9.263	0.006274	0.207	0.005	0.00063	0.001	0.378	0.029	0.00006	0.052	1.245	0.048	0.011	1.06	0.001688	0.001621	0.012	0
169	Healthy	normozoospermia	CC	0.665	126.446	10.868	8.829	3.287	61	36.2	1764	321	0.017	532	8.032	0.005964	0.195	0.004	0.002466	0	0.32	0.023	0.000201	0.017	1.419	0.051	0.018	0.981	0.002615	0.000405	0.01	0
170	Infertile	**cryptozoospermia**		0.744	17.518	8.321	10.867	1.343	61.4	39.3	1959	368	0.018	597	7.569	0.006382	0.185	0.002	0.000365	0	0.358	0.028	0.000124	0.022	1.336	0.077	0.009	0.98	0.001901	0.002276	0.01	0
171	Healthy	normozoospermia	TT	0.343	39.816	7.811	0.679	2.176	62.1	38.2	1737	356	0.018	536	8.738	0.006	0.222	0.003	0.000488	0	0.382	0.023	0.000135	0.02	1.004	0.049	0.01	0.936	0.002918	0.000371	0.013	0
173	Infertile	**asthenozoospermia**																														
174	Healthy	normozoospermia	CC	0.857	80.802	5.264	9.339	3.287	59	40.3	1914	364	0.021	609	10.317	0.005514	0.232	0.003	0.002556	0	0.418	0.02	0.000195	0.019	1.133	0.051	0.01	0.959	0.003011	0.000478	0.011	0
175	Infertile	**Combined**	CC	0.56	23.515	3.227	11.376	1.157	57.4	40.1	1739	363	0.022	563	7.128	0.005428	0.197	0.003	0.002501	0	0.407	0.015	0.000196	0.024	1.024	0.05	0.012	0.964	0.005553	0.002927	0.012	0
176	Healthy	normozoospermia		0.784	15.888	8.321	4.415	2.546	116.8	31.7	1815	281	0.105	441	20.175	0.012477	1.419	0.04	0.015421	0.001	1.162	0.111	0.000927	0.081	7.393	0.521	0.096	9.764	0.01372	0.002749	0.094	0
177	Infertile	**Combined**		0.499	33.761	11.377	2.717	2.824	51.7	35.2	1827	342	0.024	551	9.037	0.005129	0.216	0.004	0.000527	0	0.432	0.055	0.000045	0.032	1.229	0.047	0.009	0.887	0.00194	0.0007	0.013	0
178	Infertile	**polyzoospermia**	TT	0.251	26.95	9.34	3.735	2.454	52.1	36.1	1698	343	0.018	569	9.267	0.004162	0.215	0.003	0.002502	0	0.399	0.031	0.00006	0.02	1.111	0.046	0.01	0.823	0.005068	0.000328	0.017	0
179	Healthy	normozoospermia	TT	0.263	41.504	4.246	1.698	3.009	68	37.5	2030	338	0.035	510	9.874	0.004965	0.21	0.003	0.003208	0	0.461	0.017	0.000163	0.02	1.445	0.05	0.037	0.854	0.001916	0.000259	0.013	0
